# A Review of Advanced Biomaterials and Cells for the Production of Bone Organoid

**DOI:** 10.1002/smsc.202300027

**Published:** 2023-07-05

**Authors:** Chao Li, Yipu Zhang, Yawei Du, Zhiyong Hou, Yingze Zhang, Wenguo Cui, Wei Chen

**Affiliations:** ^1^ Department of Orthopaedic Surgery Key Laboratory of Biomechanics of Hebei Province Orthopaedic Research Institution of Hebei Province NHC Key Laboratory of Intelligent Orthopaedic Equipment The Third Hospital of Hebei Medical University No.139 Ziqiang Road Shijiazhuang 050051 P. R. China; ^2^ Department of Orthopaedics Shanghai Key Laboratory for Prevention and Treatment of Bone and Joint Diseases Shanghai Institute of Traumatology and Orthopaedics Ruijin Hospital Shanghai Jiao Tong University School of Medicine 197 Ruijin 2nd Road Shanghai 200025 P. R. China

**Keywords:** bone organoids, orthopedic disease models, regenerative medicine, scaffold materials

## Abstract

Rapid advancements in traditional bone tissue engineering have led to innovation in bone repair models and the resolution of insurmountable clinical issues like graft scarcity. The pathophysiological process of treating bone disease, however, is a multidimensional and multimodal regenerative regulatory mechanism that includes numerous immune, inflammatory, or metabolic responses related to the graft or the organism itself. Based on a 3D in vitro cell culture system that is remarkably identical to the body's bone tissue, the bone organoid is a biomimicking bone organ environment. It can accurately mimic the actual repair and regeneration condition in vivo because it shares the same physiological function, structure, morphology, and metabolic process as endogenous bone tissue. As a disruptive regenerative medicine technology, it has wide application prospects in the fields of organ development, gene editing, disease modeling, and precision therapy. Herein, the development process and physiological basis of different cell‐based bone organoids are reviewed, the current status of the application of different materials, cells, and construction methods for building bone organoids is described, and the prospects and challenges for the development of bone organoids in future medical fields is discussed.

## Graphical abstract

Based on a 3D in vitro cell culture system that is remarkably identical to the body's bone tissue, the bone organoid is a bionic bone organ environment. The development process and physiological basis of different cell‐based bone organoids is presented, and the current status of the application of different materials, cells, and construction methods for building bone organoids is described.

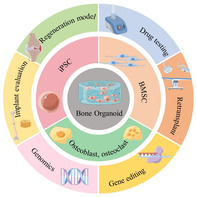

## 1 Introduction

Orthopedic diseases, as one of the most common nonlethal diseases, often have a poor prognosis due to multiple complications and impose a significant economic burden on the individual and society. The complex molecular mechanisms and diverse physiological microenvironments are two important avenues for researchers to pursue to discover new treatment modalities for orthopedic diseases.^[^ ^1^ ^]^ Various growth or regulatory factors in the cellular microenvironment strictly control the production and migration of all bone cells, inflammatory cells, immune cells, and others during the pathological repair process.^[^ ^2^ ^]^ In orthopedic diseases, rational simulation of pathophysiological processes such as intercellular communication, immune regulatory processes, or microenvironmental inflammatory factor levels can aid in the exploration of higher quality therapeutic evidence. As a result, various cellular and animal experiments are frequently used to assess the preclinical effectiveness of various drugs, biologics, or biomaterials.^[^ ^3^ ^]^ Traditional cellular assays use disease‐related cell lines or primary cells to assess drug toxicity, resistance, efficacy, and effects on cell differentiation, value‐added, and migration. Although the mechanism of action and pharmacological properties of drugs can be studied at the cellular level, the data are limited because the monolayer 2D culture differs significantly from a real complex 3D system.^[^ ^4^ ^]^ When compared to in vitro cellular experiments, animal experiments, as advanced preclinical prediction models, are more reflective of pathogenesis in a multisystem environment.^[^ ^5^ ^]^

In vivo experiments serve as a bridge between in vitro and clinical trials, providing evidence for the efficacy and safety testing of drugs or biologics above the cellular level of research. As common orthopedic conditions, bone defects (fractures),^[^ ^6^, ^7^ ^]^ osteoarthritis,^[^ ^8^, ^9^ ^]^ osteoporosis,^[^ ^10^ ^]^ and bone tumors^[^ ^11^ ^]^ have high morbidity, disability, and recovery time. To some extent, the proliferation of cutting‐edge treatment modalities has overcome these drawbacks, but there is still a scarcity of validated in vivo assessment data. The distal tibia and femur of rabbits are used in the traditional bone defect modeling approach, and a circular defect of about 5 mm in diameter is made at the corresponding site.^[^ ^10^, ^11^ ^]^ Alternatively, a dental drill was used to make an 8 mm defect in the rat cranial region.^[^ ^12^ ^]^ Despite their small size and ease of manipulation, rabbits and rats face the fundamental problem of bone anatomy differences from humans. Surgery, such as medial meniscectomy and anterior cruciate ligament resection, is frequently used to induce osteoarthritis in animal models.^[^ ^13^ ^]^ Differences in anatomy and biomechanics, on the other hand, allow for different interactions in the intra‐articular microenvironment of common animal models, influencing the final pathological progression.^[^ ^14^ ^]^ Ovariectomy‐induced osteoporosis animal models exhibit cancellous bone loss, increased bone resorption in the endosteum, and medullary cavity enlargement.^[^ ^15^, ^16^ ^]^ However, cancellous bone loss is uncommon in rodents, which is quite different from osteoporosis in humans. The induction of carcinogenic substances such as radioactive elements or vinyl chloride is used in bone tumor models.^[^ ^17^ ^]^ However, bone is not the only organ that carcinogens can affect. They can also affect endocrine and immune regulatory processes, making it more difficult to understand the therapeutic mechanisms of bone tumor models. A single animal model simplifies the human disease process, and treatment outcomes do not fully reflect the human situation. As a result, a preclinical alternative testing platform to better predict human response is urgently needed.^[^ ^18^, ^19^ ^]^

**Figure 1:**
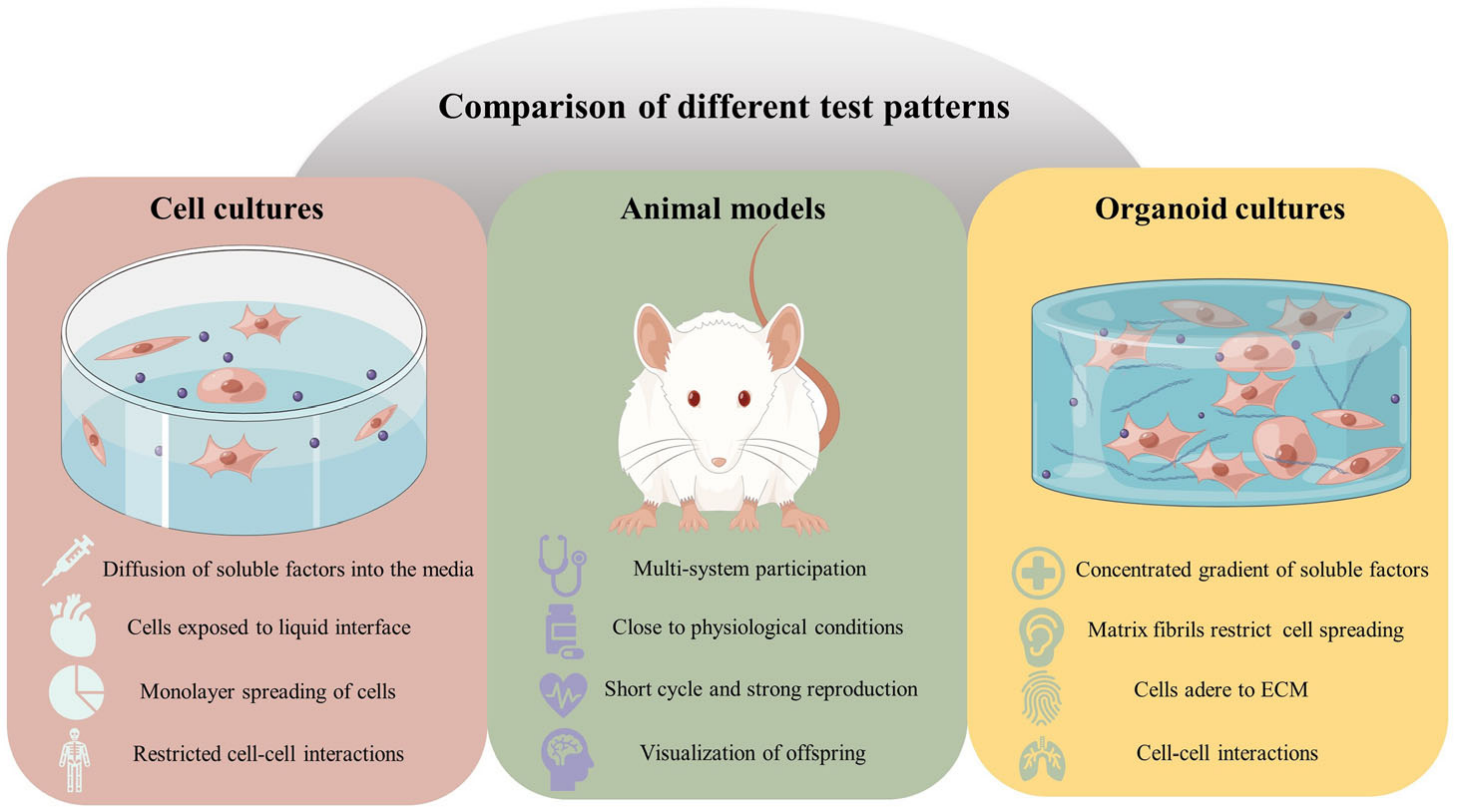
Comparison of different test patterns. Created using Figdraw.com.

Three‐dimensional cell culture (TDCC) refers to the in vitro coculture of carriers of different materials with a 3D structure and different types of cells, so that the cells can migrate and grow within the 3D spatial structure of the carriers, forming a 3D cell‐carrier complex. 3D cell culture technology is a technique between monolayer cell culture and animal experimentation that can maximally simulate the in vivo environment and demonstrate the advantages of intuitive and controllable cell culture conditions. Unlike cellular and animal models, the bone organoid is a biomimicking bone organ environment based on a 3D in vitro cell culture system highly similar to the body's bone tissue^[^ ^20^ ^]^ (**Figure** **1**). The 3D in vitro culture system can replicate the complex spatial morphology of in vivo tissues while exhibiting similar physiological and chemical reactions of the corresponding tissues or organs. Bone organoids not only contain multiple cell types, such as osteoblasts, osteoclasts, and mesenchymal stem cells, but also provide a more comprehensive view of the interactions and spatial patterns between different cells, cells and matrices.^[^ ^21^ ^]^ Bone organoids with multiple cell types can better mimic the physiopathological processes of human bone tissue, and the self‐renewal, migration, differentiation, and mutation processes of 3D cell clusters also provide the basic conditions for bone organoids. In this regard, the phenotype of stem cells is inextricably linked to the development of the organoid. Stem cells can differentiate into homogeneous populations of various cell types under 2D or 3D culture conditions. The unlimited capacity for cell renewal and value addition confers the special ability to generate daughter cells or organ‐specific cells genotypically identical to their own. Stem cells have extremely promising applications and important potential for development in the fields of orthopedic disease treatment, organoid biology, and drug testing. Bone defects, as one of the most common types of orthopedic diseases, were also the first models for the application of stem cell therapy. If stem cells are used to construct bone organoids, they will make a great contribution to drug screening and alternative treatment.

In addition to stem cells, functional cells, extracellular matrix (ECM), cytokines, multiple immune cells, neural cells, and inflammatory cells are widely dispersed in bone pathological tissues.^[^ ^22^ ^]^ Although bone organoids have unique cellular characteristics, their maturation process is accompanied by microenvironmental requirements. Among them, bone organoids mainly rely on artificial ECM to refine the hierarchical structure of natural bone tissue. Existing bone organoids or other organoids often rely on commercial matrix gels. Although matrigels have physicochemical properties that are closer to the real human ECM, uncontrollable factors of batch‐to‐batch stability limit their widespread use. In contrast, engineered hydrogel materials that can be conditioned and modified are more conducive to the culture of bone organoids. Collagen or hyaluronic acid hydrogels, which have the same physicochemical properties as extracellular mesenchyme, are the materials of choice for bone organoid model construction.^[23a,23b]^ Hydrogels are made of cross‐linked hydrophilic polymers that maintain a 3D mesh structure while releasing cytokines, absorbing water, stabilizing degradation, and enhancing cell viability.^[24a,24b]^ The choice of bone organoid matrix material determines the cell's survival, morphology, and function. The development of a range of 3D hydrogel materials can better mimic orthopedic disease properties and influence cellular behavior. The highly systematic and integrated construction criteria have led to a large number of applications of bone organoids for the study of bone regeneration, bone homeostasis, and bone modulation. And the combination of advanced materials and stem cells will further promote the continued development of bone organoids.

**Figure 2:**
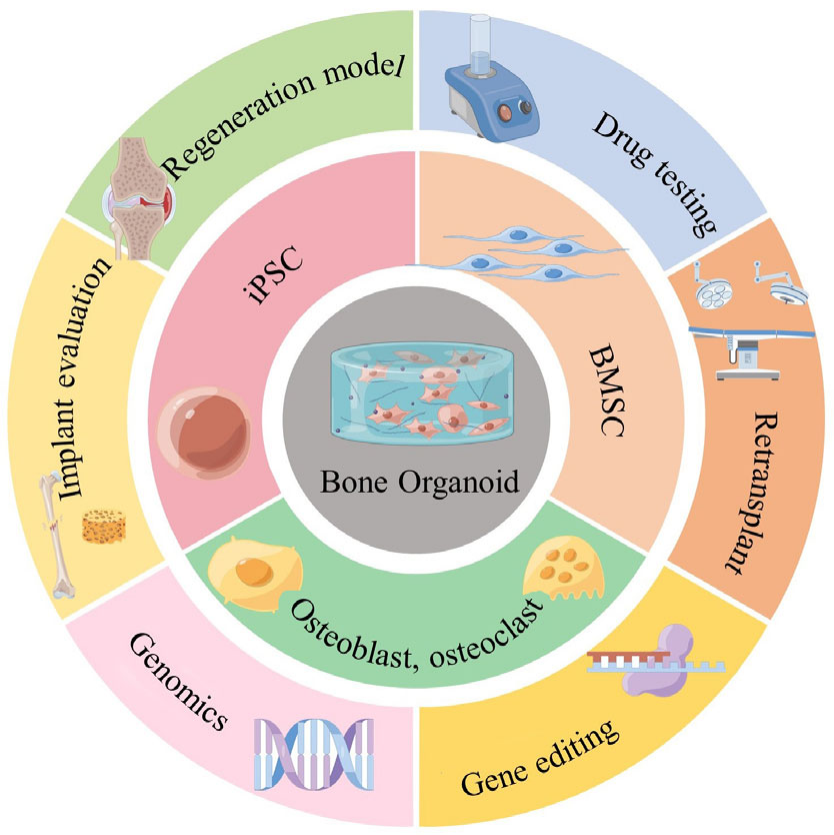
Schematic diagram of bone organoid cell composition and application direction. Created using Figdraw.com.

Bone organoids use bioactive materials as a scaffold to assemble stem cells and various types of functional cells in the bone regeneration microenvironment into biomimicking bone tissue that resembles the spatial structure of bone. Bone organoids not only have the spatial structure and specific functions of bone tissues but also can mimic bone pathophysiological processes, which is a good model for the study of bone regulation and bone regeneration mechanisms.^[^ ^25^ ^]^ Different bone organoid models have some common characteristics, such as: 1) they are composed of multiple characteristic cells of donor bone tissue; 2) they have the physiological functions of bone tissue; and 3) they have similar cell or tissue arrangement morphology with bone tissue. Simply put, bone organoids are an upgraded version of the traditional cell culture monolayer model. It not only reflects the heterogeneity of cells but also provides a higher degree of morphological characteristics. A comparison of organoids with cellular and animal experiments is shown in **Table** **1**. The advantages of bone organoids in maintaining genomic stability, reducing experimental complexity, and adapting to new technologies make them powerful tools for bone disease modeling and drug testing.^[^ ^26^ ^]^ Materials and stem cells, two of the most important components of bone organoids, often control the maturation process and functional properties of bone organoids. In this article, we present a review of the construction and scope of application of bone analogous organ models, systematically describing the pathophysiological basis of bone disease models and the bone microenvironment. We highlight the types and limitations of biomaterials related to bone organoids. Then, we listed the functions and characteristics of different sources of stem cells. Finally, we summarize the main roles and application scope of bone organoids in the construction of bone disease models and discuss the direction of their subsequent development (**Figure** **2**).

**Table 1:** Comparison of cell cultures, animal models and organoid cultures

	Cell cultures	Animal models	Organoid cultures
Incubation time	Short	Long	Normal
Success rate	Medium	Low	High
Physiologic representation	Limited	Physiologic	Semiphysiologic
High‐throughput screening	High	Low	High
Manipulability	Excellent	Limited	Good
Genome editing	Permit	Permit	Permit
Complexity	Simple	Normal	Complex
Cost	Low	High	Normal
Vascularization	No	Yes	No
Immune system	No	Yes	No

## 2 Relevant Bone Properties for the Generation of Bone Organoids

### 2.1 Key Structures of the Skeletal System

Bone is mainly composed of osteocytes, collagen fibers, and cellular matrix, which play an irreplaceable role in the metabolic activities and growth and development of the human body.^[^ ^27^ ^]^ According to its different morphologies, human bone can be divided into long, short, flat, and irregular bones.^[^ ^28^ ^]^ The middle bone of the lone bone is dense and connects the two end epiphyses, called the backbone. The bone stem contains a cavity, which is where the bone marrow is stored and is also called the marrow cavity.^[^ ^29^ ^]^ The wrist bone of the hand and the tarsus of the foot are the main short bones of the body.^[^ ^30^, ^31^ ^]^ The flat bones often serve as the walls of the bone cavity and mainly protect and support the organs within the cavity.^[^ ^32^ ^]^ Irregular bones such as vertebrae, mandibles, and sieve bones have no fixed morphology and together form the remainder of the human skeleton. The function and morphology of bone are inextricably linked and mutually constrained. Different bony structures have special motor functions or load‐bearing significance. In particular, the parts close to the joint cavity need to be considered as multicellular and multitissue together. The presence or absence of overlying articular cartilage, the need for support, and the structural integrity of the closure are important guiding parameters for the culture of bone organoids.

**Figure 3:**
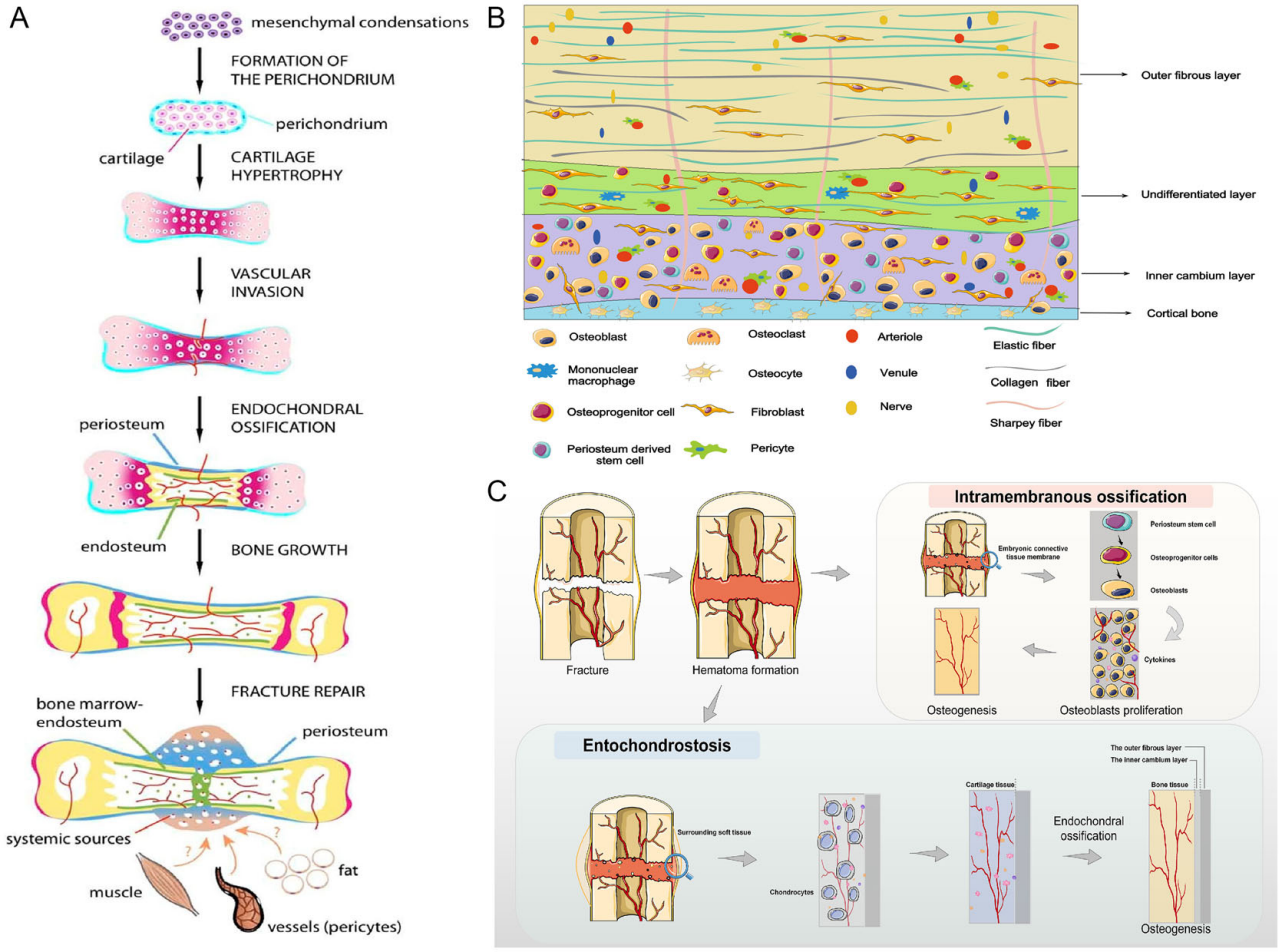
Osteotropic effect of periosteum. A) Development of the periosteum and its role in bone repair. Reproduced with permission.^[^ ^34^ ^]^ Copyright 2012, Orthopaedic Research Society, published by Wiley. B) Histological structure of the periosteum. C) Intramembranous osteogenesis and endogenous cartilage formation are the two primary types of periosteal osteogenesis. Reproduced with permission.^[^ ^36^ ^]^ Copyright 2022, Elsevier.

**Figure 4:**
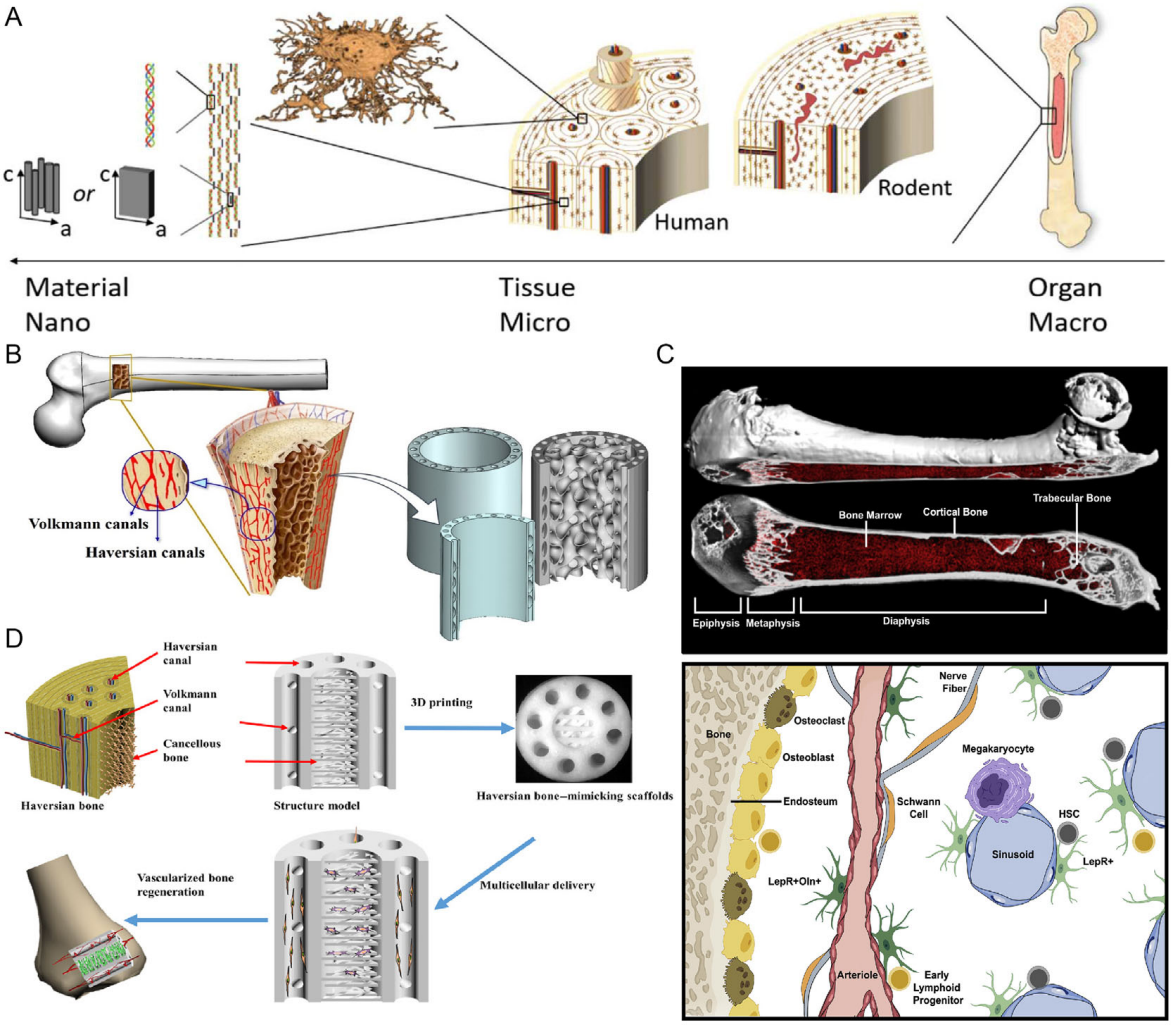
Bone multilayer structure and bone unit microenvironment. A) Bone structure on different scales. Reproduced with permission.^[^ ^27^ ^]^ Copyright 2022, International Union of Crystallography. B) Biomimetic design of implants for cortical and cancellous bone. Reproduced with permission.^[^ ^37^ ^]^ Copyright 2022, Elsevier. C) Bone marrow anatomy and hematopoietic microenvironment. Reproduced with permission.^[^ ^43^ ^]^ Copyright 2021, Elsevier. D) Simulated bone unit tissue engineering scaffold. Reproduced with permission.^[^ ^48^ ^]^ Copyright 2020, The Authors, published by American Association for the Advancement of Science.

In addition to its daily load‐bearing function, the outstanding motor function and repair and regeneration capacity are the main reasons why bone is one of the most important organs of the human body. The periosteum, as the most regenerative potential structure in bone, consists of an outer fibrous layer and an inner osteogenic layer^[^ ^33^, ^34^ ^]^ (**Figure** **3**A). Fibroblasts, collagen, blood vessels, and nerves are the main components of the fibrous layer, maintaining its nutritional and protective role. The inner layer is highly cellular and contains a variety of osteogenic‐associated cells, such as mesenchymal progenitor cells, osteogenic progenitor cells, and osteoblasts (Figure 3B). The periosteum is in contact with the cortical surface of the bone, which thickens during fracture defects, and osteogenic processes occur strongly with periosteal‐derived progenitor cells^[^ ^35^, ^36^ ^]^ (Figure 3C). Bone is divided into cortical bone composed of bone lamella and cancellous bone composed of bone trabeculae^[^ ^37^, ^38^, ^39^, ^40^, ^41^ ^]^ (**Figure** **4**A,B). As the main site of hematopoiesis, the small blood vessels of the bone marrow pass through the bone in close connection with the surrounding reticulocytes.^[^ ^42^ ^]^ Hematopoietic cells and other cells such as adipocytes, osteoblasts, and fibroblasts are all present in the bone marrow and form the intramedullary hematopoietic microenvironment together with other regional tissues^[^ ^43^ ^]^ (Figure 4C).

Compared to the repairing effect of natural bone, the in vitro simulation of bone organs also needs to refer to the natural bone structure and microenvironmental components. Different bone densities, marrow cavity composition, and periosteal layers often determine the final repair and regeneration effect. There are some differences in the arrangement and number of bone lamella in cancellous and cortical bone. Similarly, there are significant spatial differences in the stem cell content of the marrow cavities of long and flat bones. The fabrication of bone organoids requires not only mimicking the natural osteogenesis process but also replicating osteogenic conditions and maintaining dynamic relationships based on osteogenesis.

The bone unit is a characteristic hierarchical structure unique to the bone. The four components of the bone unit (osteocyte, Haversian canal, Haversian lamellae, and lacuna‐canalicular network) lay the foundation for the supportive role of bone.^[^ ^44^ ^]^ The Haversian canal contains nerves and blood vessels that provide nutrition to osteoblasts while undertaking the task of information transfer and material exchange. Osteocyte dendrites extend to the bone surface through the luminal canal network and Haversian canals to perform mechanosensory transmission tasks.^[^ ^45^ ^]^ As mentioned earlier, the luminal space where the osteocytes are located and the tubules where the dendrites are located together constitute the luminal canal network. The luminal canal network contains flowing fluid between the osteocytes and the bone matrix, which facilitates the material transfer and signal exchange, and also indirectly enables stress sensing and transmission.^[^ ^46^ ^]^ The central canal is surrounded by 4–20 layers of concentrically arranged bone lamella. The fibrous layers in the bone lamella determine the degree of bone anisotropy and give the bone a good resistance to compression and tension.^[^ ^47^ ^]^ The complexity of the bone unit hierarchy and the high standard of mechanical properties required have also promoted the innovation of next‐generation tissue engineering scaffolds. Zhang et al.^[^ ^48^ ^]^ used 3D printing technology to prepare a mock scaffold with a complete hierarchical structure of the Haversian system, showing great potential for inducing osteogenic and angiogenic differentiation (Figure 4D).

The bone unit, as the most basic and important primary structure in the osteogenic system, is a strong guide for the construction of bone organoids. The transverse and longitudinal canals are the conduits for the transfer of nutrients and metabolic waste. If similar canal structures can be made in bone organoids, it will facilitate the exchange of information between cells. The bone unit is also a critical structure for the evolution of a single functional cell into a tissue. The basic formation of bone units in vitro reflects the successful construction of bone organoids.

### 2.2 Steps in Bone Regeneration

Osteogenesis is a complex and continuous process that encompasses changes in all phases of histology and cytology. Traumatic repair is one of the most important causes of late osteogenesis and involves communication and reactions between multiple cells, tissues, and organs. In general, bone defects lead to rupture and bleeding of the bone marrow, periosteum, and surrounding blood vessels, forming a hematoma. Within 6–8 h thereafter, products released from the ischemic and necrotic cells cause local capillary dilation, plasma exudation, and inflammatory cell infiltration. Approximately 2 weeks later, severed osteocytes, osteoblasts, and bone matrix release insulin growth‐like factor (IGF), basic fibroblast growth factor (bFGF), platelet‐derived growth factor (PDGF), and beta transforming growth factor (TGF‐β) into the surrounding microenvironment. Numerous growth factors promote MSC aggregation, proliferation, and vascular proliferation while stimulating osteogenic differentiation.^[^ ^49^ ^]^ In the periosteum compartment, periosteum‐derived cells (PDCs) are more directly involved in bone repair by forming cartilage and bone in the callus. The proliferative phase is then associated with the formation of the cartilaginous fracture callus, where skeletogenic stem cells proliferate to form a soft callus. The soft callus is then mineralized by endochondral ossification to form a hard callus. The bone matrix gradually settles and the primitive fibrous junctions continue to ossify, forming new bone. The new bone is continuously calcified and strengthened by the promotion of trace elements and hormones. The mechanical strength is also sufficient to resist shear or contraction forces.^[^ ^50^ ^]^ Different cytokines have different regulatory effects on the biological process of osteogenesis. The rational addition of relevant growth factors contributes to the maturation and development of bone organoids.

**Figure 5:**
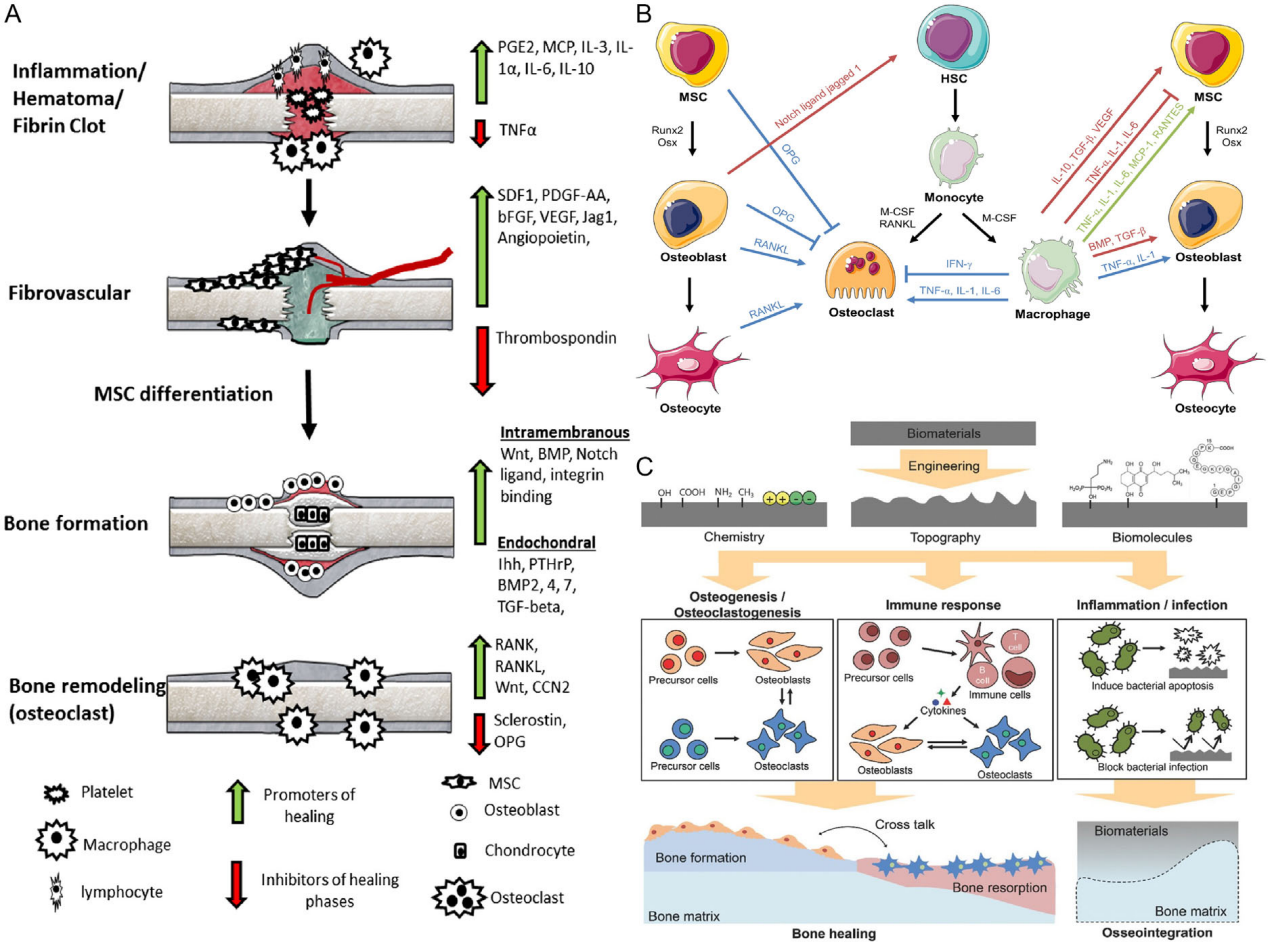
The inflammatory repair phase has a crucial regulatory role in bone repair. A) Signaling factors that produce positive and negative effects at each stage of fracture healing. Arrows: green (positive effect) and red (negative effect). Reproduced with permission.^[^ ^51^ ^]^ Copyright 2015, Elsevier. B) Communication and regulation between inflammatory cells and osteoprogenitor cells. Reproduced with permission.^[^ ^55^ ^]^ Copyright 2016, Elsevier. C) Tissue engineering materials for various biomodulation processes. Reproduced with permission.^[^ ^56^ ^]^ Copyright 2018, Wiley‐VCH.

As previously mentioned, the inflammatory repair phase has a crucial regulatory role in bone repair. Different types of inflammatory cells such as macrophages, lymphocytes, and neutrophils secrete different signaling molecules, which affect the processes of osteogenic and chondrogenic differentiation, angiogenesis, and stem cell recruitment through different signaling pathways^[^ ^51^ ^]^ (**Figure** **5**A). And the final bone repair effect is highly dependent on the initial inflammatory phase.^[^ ^52^ ^]^ The hematoma serves as an aggregation point for various inflammatory cells and inflammatory cytokines, which first initiates the inflammatory cascade response.^[^ ^53^ ^]^ Macrophages and other cells not only phagocytose cellular debris and necrotic bone tissue but also recruit mesenchymal stem cells and fibroblasts to move toward the defect site. At the histological level, the inner layer of the periosteum contains osteogenic progenitor cells, chondrogenic progenitor cells, and endothelial cells, which provide the cellular basis for bone and cartilage formation and vascularization. In turn, the migration, value‐added, and differentiation of these cells at all stages are regulated by signaling factors.^[^ ^54^ ^]^ The interregulation process of macrophage–osteoclast and mesenchymal stem cell–osteoblast is a microcosm of the whole inflammatory response and osteogenic repair process interfused and interacting with each other^[^ ^55^ ^]^ (Figure 5B). Inspired by this, biomaterials can provide structural support, mechanical support, regulatory drugs, and molecular carriers for damaged tissues and have a wide range of applications in the field of tissue engineering. Current biomaterials can be involved in various biological processes, such as immune response, inflammation regulation, osteogenic differentiation, and osteoclast formation (Figure 5C). By implanting biologically active materials into the body, various types of biological signals are triggered to accomplish specific material–tissue interactions.^[^ ^56^ ^]^

## 3 Advanced Biomaterials Mimic Bone Mineralization Matrix

### 3.1 Inspiration for Mineralization of Bone Organoid

As an effective model to simulate real mechanisms of osteogenesis, resorption, mineralization, and remodeling, bone organoids should be constructed strictly according to bone morphology. However, only mineralized bone has unique biomechanical properties to accomplish tasks such as support, load‐bearing, and strain.^[^ ^57^ ^]^ Bone mineralization refers to the process of inorganic deposition of elements such as calcium and phosphorus accomplished under the regulation of hormones or cytokines. The past research task bone mineralization is a passive process. After decades of pushing the boundaries, scientists have come to realize that bone mineralization is a programmed process under the regulation of multiple factors. The level of inorganic components, the synthesis of extracellular matrix (ECM), and the formation of mineralized crystals are all controlled and influenced by multiple genetic pathways.^[^ ^58^ ^]^

**Figure 6:**
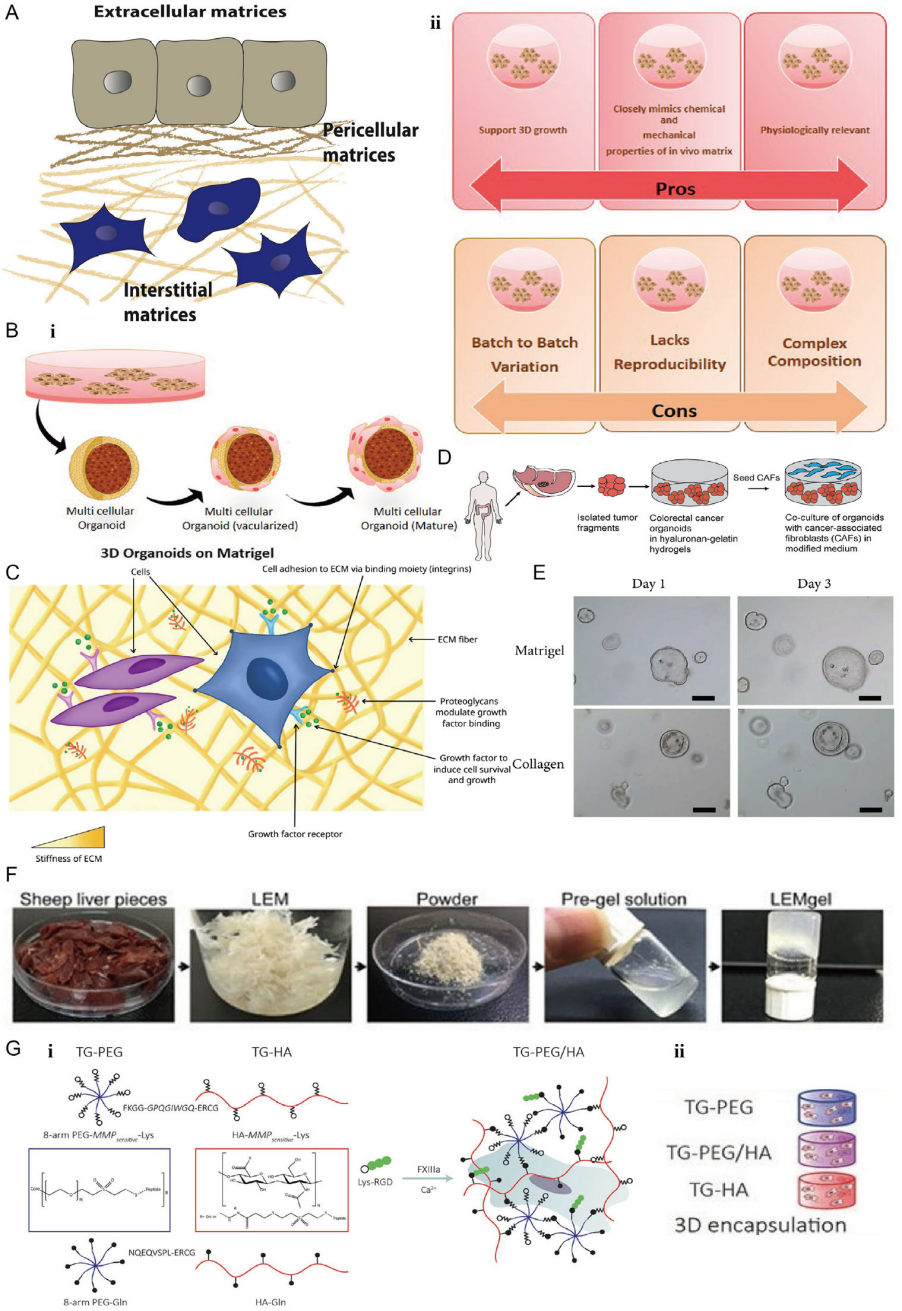
Advanced biomaterials mimic bone mineralization matrix. A) Structure of the extracellular matrix. Reproduced with permission.^[^ ^59^ ^]^ Copyright 2016, Elsevier. B) (i) Schematic diagram of matrix gel‐based organoid formation. (ii–iii) Pros and cons of choosing matrix gels as extracellular matrix during organoid formation. Reproduced with permission.^[^ ^67^ ^]^ Copyright 2021, Elsevier. C) Cells in organoid are surrounded by other cells, ECM proteins and proteoglycans and growth factors. Reproduced under the terms of the CC‐BY Creative Commons Attribution 4.0 International license (https://creativecommons.org/licenses/by/4.0).^[^ ^68^ ^]^ Copyright 2021, The Authors, published by Springer Nature. D) Organoids derived from patients with colorectal cancer are composed of 3D hyaluronic acid–gelatin hydrogel. Reproduced with permission.^[^ ^69^ ^]^ Copyright 2021, Elsevier. E) Images of mouse colonic organoids in matrigel and collagen matrix culture system. Reproduced under the terms of the CC‐BY Creative Commons Attribution 4.0 International license (https://creativecommons.org/licenses/by/4.0).^[^ ^70^ ^]^ Copyright 2019, The Authors, published by Hindawi. F) Schematic diagram of preparation of decellularized liver‐derived hydrogel. Reproduced with permission.^[^ ^73^ ^]^ Copyright 2017, Wiley. G) (i) Polyethylene glycol/hyaluronic acid hybrid hydrogel. (ii) Diagram of hydrogel embedding. Reproduced with permission.^[^ ^78^ ^]^ Copyright 2020, Wiley‐VCH.

The process of bone mineralization in vitro in organoids is inseparable from the building of ECM, a complex network of multiple macromolecules surrounding cells (**Figure** **6**A). ECM and cells are interspersed and interconnected, enabling the formation of more complex and functional tissues and organs.^[^ ^59^ ^]^ ECM as a noncellular component not only serves as a scaffold for cellular embedding but also is responsible for regulating a variety of cellular activities including value addition, migration, and differentiation.^[^ ^60^, ^61^ ^]^ The bone ECM is mainly composed of collagen, elastin, proteoglycan, and glycosaminoglycan. Among them, collagen is not easily degradable and is the most abundant component of the ECM. Collagen mainly provides the bone matrix with the ability to resist tension. Collagen is secreted by osteoblasts, chondrocytes, and fibroblasts. The cells in the bone microenvironment and the ECM work together to have a critical impact on the development and progression of various bone diseases.^[^ ^62^ ^]^ ECM contains a large number of signaling molecules that are actively involved in controlling cell growth, polarity, shape, migration, and metabolic activities. The development of bone organoids depends on the support and transmission functions of the ECM. Matrix derivatives derived from different substances confer an excellent capacity for the regeneration and maturation of bone organoids in different directions (**Table** **2**).

**Table 2:** Application of different materials for organoid construction

	Advantages	Disadvantages	Literature
Matrigel	Rich in components	Poor reproducibility	[64, 66, 67]
Natural hydrogel	High biocompatibility	Lack of mechanical properties	[68]
Synthetic hydrogel	Controlled physical and chemical properties	Complex preparation	[75]

### 3.2 Biomaterials used in Bone Organoid Generation

Biomimicking‐constructed bone organoids are based on models created by 3D in vitro cell culture systems. Cell clumps or cell spheres formed solely by multicellular agglutination often have defects in morphological maintenance or functional transfer. Bioactive materials are becoming an important component in the construction of bone organoids due to their good biocompatibility and excellent physicochemical properties

### 3.3 Matrigel for Bone Organoid

Matrigel, the most common ECM‐derived material, is commonly used as a biomimicking 3D culture environment.^[^ ^63^ ^]^ Matrigel originated from a chance discovery more than 40 years ago. At that time, a special tumor that secretes ECM was identified in mice and named Engelbreth–Holm–Swarm (EHS). Matrix gum, as an extract of EHS, mainly contains laminin, type IV collagen, heparin sulfate proteoglycan, entactin, transforming growth factor, fibroblast growth factor, and matrix metalloproteinase.^[^ ^64^ ^]^ During the preparation process, matrigel undergoes gelation and forms a swollen hydrogel network. Matrigel provides a carrier for cell attachment due to their rich content of growth factors and collagen. Brain organoids constructed using matrigel grow larger and faster in suspension compared to the culture model without matrigel.^[^ ^65^ ^]^

Currently, matrigel is allowed to encapsulate cells in the assembly of organoids. However, animal‐derived matrigel is expensive, variable in composition and presence of xenobiotic contaminants, and strictly limited in applicability.^[^ ^66^ ^]^ In addition, there is a great variation between batches of matrigel, making personalization more difficult to achieve.^[^ ^67^ ^]^ Moreover, simple matrigel is prone to degradation during the development of bone organoids and is insufficient to achieve standard levels of mechanical support (Figure 6B).

### 3.4 Biochemically Natural Hydrogels for Bone Organoid Engineering

Natural hydrogels are partly constituents of ECM and partly derivatives of ECM. Therefore, natural hydrogels are extremely similar in physicochemical properties and biological activity compared to human ECM. Because of this, natural hydrogels are one of the most excellent and ideal materials suitable for cell growth and development. Polysaccharides and proteins such as collagen, hyaluronic acid, gelatin, chitosan, sodium alginate, and heparin are the most classical natural hydrogels. As a carrier of cell adhesion, natural hydrogels not only communicate with cells, but also diffuse cytokines and provide a stable physiological microenvironment^[^ ^68^ ^]^ (Figure 6C).

Luo et al.^[^ ^69^ ^]^ reported a patient‐derived organoid from colorectal cancer. The organoid was composed of hyaluronic acid and gelatin hydrogel (Figure 6D). The team evaluated the stiffness, suitability, and stability of the hydrogel, and all results demonstrated that the hyaluronic acid–gelatin hydrogel matrix was able to maintain the proliferation capacity of the organoid. Jee et al.^[^ ^70^ ^]^ attempted to develop a new collagen matrix for mouse colon organoid culture. The current study found that collagen‐based organoids were able to grow and develop successfully and had similar properties to those cultured in matrix gels (Figure 6E). Collagen could replace animal‐derived matrix gels to help enhance the efficacy of organoids for the treatment of trauma. Wang et al.^[^ ^71^ ^]^ synthesized composite hydrogels with high‐throughput properties using fibrin hydrogels as the core and an outer coating of sodium alginate–chitosan shell. Subsequently, induced pluripotent stem cells (iPSCs) were added to complete the differentiation and culture of liver organoids. The mature liver organoids not only retain the liver‐specific functions but also continue to grow and maintain a high biological activity.

Although natural hydrogels have the advantages of high water content and high biocompatibility, their relative lack of stability and mechanical properties is also of concern. Although the use of cross‐linking agents can improve the mechanical properties of hydrogels, chemical cross‐linking agents have also been reported to be potentially unwanted toxic to surrounding or encapsulated cells. For example, unbound free aldehyde groups are mainly responsible for the toxic effect of glutaraldehyde cross‐linked scaffolds.

Recently, the use of decellularized matrices has also been gradually applied to the construction of organoids.^[^ ^72^ ^]^ The decellularized matrix is all components of the tissue except for the cells. The decellularized matrix is processed to remove antigenic components that can cause immune rejection while leaving intact the 3D spatial structure of the ECM and some growth factors that are important for cell differentiation. Saheli et al.^[^ ^73^ ^]^ used decellularized matrix hydrogels to construct liver organoids. This study evaluated various physicochemical properties of decellularized matrix hydrogels and demonstrated that they could enhance the functional activity of liver organoids (Figure 6F). Organoids cultured in the presence of this decellularized matrix hydrogels manifested the epithelial phenotype of hepatocytes with higher cell viability and significant upregulation of hepatocyte‐specific transcripts and functions compared to hydrogel‐free organoids. Kim et al.^[^ ^74^ ^]^ investigated the feasibility of decellularized matrix hydrogels in the construction of gastrointestinal organoids. It was demonstrated that the gastrointestinal exo‐matrix hydrogel not only provided a good gastrointestinal simulation environment but also realized the long‐term culture of the organoid. Together, the orchestration of gastrointestinal tissue‐specific matrisome and nonmatrisome core proteins enables the reconstruction of native gastrointestinal tissue‐mimetic microenvironments to support the growth and development of gastrointestinal organoids.

### 3.5 Biochemically Synthetic Hydrogels for Bone Organoid Engineering

The limitations of matrigel and natural hydrogels have led to a search for alternatives with better properties and wider applicability. Synthetic hydrogels are composed of hydrophilic polymers including poly(ethylene glycol), poly(vinyl alcohol), poly(lactic acid), and polyacrylamide. The greatest advantage of synthetic hydrogels is that their physicochemical properties can be controlled.^[^ ^75^ ^]^ Modifying or modifying the matrix metalloproteinase binding site during the synthesis process can achieve the goal of regulating the rate of hydrogel degradation. Similarly, using different preparation processes, such as electrostatic spinning^[^ ^76^ ^]^ or microfluidics,^[^ ^77^ ^]^ can change the shape and size of the hydrogel or organoid. In addition, researchers can also replicate the composition or functional properties of organoids in vitro by imposing conditions that are specific to the natural organ or microenvironment.

Vallmajo‐Martin et al.^[^ ^78^ ^]^ seamlessly combined polyethylene glycol and hyaluronic acid hydrogels. It was further assembled into bone marrow organoids by the properties of polyethylene glycol and hyaluronic acid (Figure 6G). The results showed that the hybridized hydrogels possessed good physicochemical properties and significantly promoted the amplification and differentiation of hematopoietic stem cells (HSCs) and bone marrow mesenchymal stem cells (BMSCs). Compared to simply changing the binding site or shape, changing the porosity of the hydrogel is also worth considering. To maximize the survival rate of lung organ transplants, Dye et al.^[^ ^79^ ^]^ used poly(ethylene glycol), poly(caprolactone), and poly(lactide‐*co*‐glycolide). The effect of the pore size of the micropores of the different materials on airway maturation and immune response was investigated, respectively. The class organoid optimized the material parameters and established a new airway disease model. It was demonstrated that the physicochemical properties of the materials can affect the function of the organoid.

## 4 Production Strategies for Generating Biomimicking Organoids using Stem Cells

**Figure 7:**
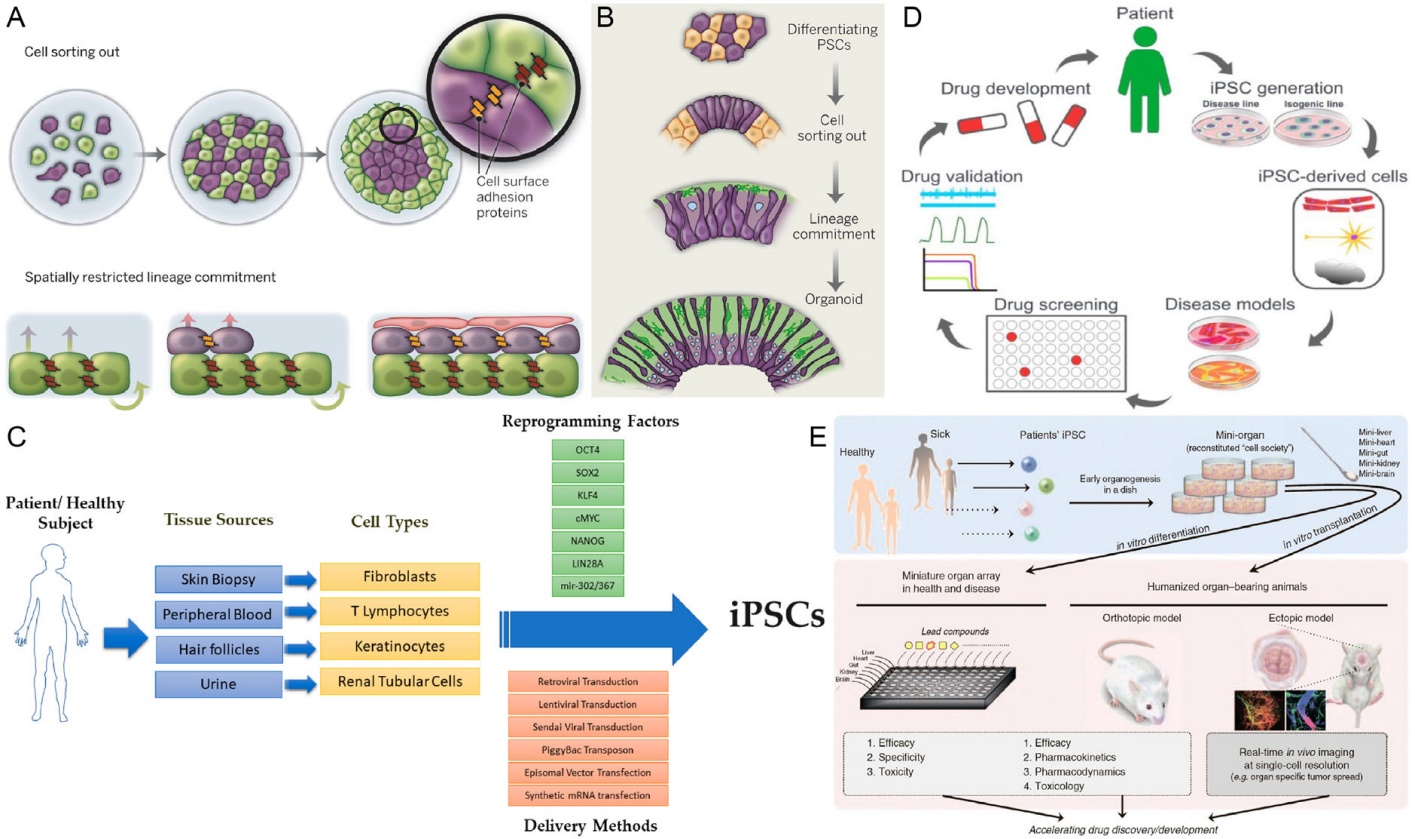
Stem cells of organoid. A) Principles of self‐organization. Different cell types (purple or green) classify themselves due to the different adhesion properties conferred by the differential expression of their different cell adhesion molecules (e.g., brown or orange bars). Progenitor cells (green) produce more differentiated progeny (purple). B) Organoid self‐organization by cell sorting and lineage commitment. A,B) Reproduced with permission.^[^ ^80^ ^]^ Copyright 2014, The American Association for the Advancement of Science. C) Schematic diagram of iPSC extraction from patients. Reproduced under the terms of the CC‐BY Creative Commons Attribution 4.0 International license (https://creativecommons.org/licenses/by/4.0).^[^ ^83^ ^]^ Copyright 2019, The Authors, published by MDPI. D) iPSC applications in drug testing and disease modeling. Reproduced under the terms of the CC‐BY Creative Commons Attribution 4.0 International license (https://creativecommons.org/licenses/by/4.0).^[^ ^87^ ^]^ Copyright 2022, The Authors, published by MDPI. E) Drug testing and pharmacological applications based on iPSC organoids. Reproduced with permission.^[^ ^89^ ^]^ Copyright 2014, American Society for Clinical Pharmacology and Therapeutics, published by Wiley.

Cells in vertebrates are capable of spontaneously reuniting to form the structure and morphology of primitive organs even if they are completely free^[^ ^80^ ^]^ (**Figure** **7**A,B). The results point to a general capacity of cells to reorganize and segregate in a process termed “cell sorting out” to form structures with much the same histogenic properties as those in vivo. This strong cellular self‐organization ability is the cellular basis for in vitro organoid construction. Organoids are mainly derived from stem cells, including embryonic stem cells (ESCs)^[^ ^81^ ^]^ and adult stem cells (ASCs).^[^ ^82^ ^]^ Most stem cells previously focused on specific types of cell populations, whereas organoid models require organ‐intact cell populations. Research on the value‐added and differentiation mechanisms associated with bone organoids is still in its infancy, and conditional directed differentiation is difficult. Nevertheless, the construction of bone organoids still starts with stem cells. How to achieve the isolation, identification and differentiation, and expansion of stem cells is the focus of current bone organoid research (**Table** **3**).

**Table 3:** Stem‐cell‐based bone and cartilage organoids

Cell type	Construction method	Main functions	Literature
iPSC	The time‐dependent nature of growth factors was exploited to adjust the culture conditions for in vitro suspension culture of human‐derived iPSCs bone organoid.	Engineered iPSCs coalesce to form glycosaminoglycan‐rich clumps after mesoderm induction. After maturation, they gradually transform into type II collagen‐rich cartilage clumps.	Tam et al.^[^ ^94^ ^]^
iPSC	Cartilage and bone organoids were constructed using human iPSCs expressing OPG‐XL and the CRISPR/Cas9 system.	Knockdown of OPG‐XL to reduce osteoclast activity. Overcoming cartilage hyperfibrosis and bone matrix hypermineralization for osteoarthritis	Rodríguez Ruiz et al.^[^ ^95^ ^]^
iPSC	TGF‐β3 and BMP2 were added sequentially in chronological order to stimulate single cell growth for organoid.	Modeling cartilage–bone interactions. Application to joint disease drug screening and patient‐specific therapy.	O’Connor et al.^[^ ^96^ ^]^ 2021
iPSC	Cartilage microtissues from two different sources can be engineered to form region‐specific cartilage implants.	Two different sources of cartilage microtissues can be engineered to form region‐specific cartilage implants. This bilayer structure mimics articular cartilage and subchondral bone, respectively.	Hall et al.^[^ ^97^ ^]^
CB‐BF	Cord blood‐derived fibroblasts form cartilage‐like organs in vitro and are later transplanted into mice.	Histomorphometric analysis of CB‐BFs and the number of hematopoietic cells isolated in bone demonstrated their excellent hematopoietic potential.	Pievani et al.^[^ ^110^ ^]^ 2017
BMSC	BMSCs loading on hydrogel microspheres by digital light processing printing technology and distribution induction technology.	BMSCs are transformed into osteocallus organoids after chondrogenesis induction, which can reproduce the osteogenesis process in cartilage.	Xie et al.^[^ ^111^ ^]^
BMSC	BMSCs were inoculated onto the collagen scaffold as a template for development. IL‐1β was later added to the culture medium to promote cartilage remodeling.	The medullary cavity of the organoid contains HSC and various types of progenitor cells that are similar to natural bone in structure and function.	Scotti et al.^[^ ^112^ ^]^
BMSC	BMSCs and nasal cartilage cells were embedded in a bilayer hydrogel. The bilayer hydrogel is made up of a TGF3 or BMP‐2 functionalized polyethylene glycol hydrogel (encapsulated BMSCs) and a nasal cartilage hydrogel.	Endochondral osteogenesis can occur in the hydrogel layer loaded with BMSCs, and cartilage tissue can be formed in the hydrogel layer of nasal cartilage.	Stüdle et al.^[^ ^113^ ^]^
BMSC	BMP‐2 scaffolded cell‐free bone organoid.	This cell‐free strategy serves as an ecological environment for the communication and exchange of stem cells and immune cells to harvest abundant autologous cells such as osteogenic, fibrogenic and hematopoietic cells.	Dai et al.^[^ ^114^ ^]^
Osteoblast, osteoclast	The model consists of a combination of osteoblastic and osteoclastic cells inoculated on the trabeculae of the femoral head.	As a multicellular organ model, it can simulate the bone loss process well. The model can effectively provide an ex vivo platform to overcome the limitations of the traditional laboratory simulation of bone microenvironment.	Iordachescu et al.^[^ ^93^ ^]^
Osteoblast	Use of an osteobiomaterial on board osteoblasts to guide structural mineralization of osteoblasts.	Repeated bone remodeling cycles maintain bone physiological homeostasis through local regulation of bone trabeculae.	Park et al.^[^ ^118^ ^]^
PDC	PDCs were used to prepare an engineered bone organoid for the regenerative treatment of long bone defects.	The height of the new bone formation and the portion bridging with the old bone are both comparable to natural long bones. Even in vitro, the natural cascade process is simulated.	Nilsson Hal et al.^[^ ^119^ ^]^

### 4.1 From Scalable iPSC to Bone Organoid

iPSCs are stem cell types that are functionally similar to natural pluripotent stem cells obtained by artificially inducing nonpluripotent cells to express a specific gene^[^ ^83^ ^]^ (Figure 7C). Takahashi et al.^[^ ^84^ ^]^ first demonstrated that differentiated cells can reprogram themselves into an embryonic‐like state by transferring nuclear contents to oocytes or fusing with ESCs. By adding four transcription factors, Oct3/4, Sox2, c‐Myc, and Klf4, to ESC culture conditions, Takahashi's team converted mouse embryonic or adult fibroblasts into iPSCs. iPSCs are easier to obtain than ESCs, which are cumbersome to culture and extract.^[^ ^85^ ^]^ iPSCs’ good multidirectional differentiation and scalability are their most important It is also the basis for the construction of in vitro organoids. Not only that, but iPSC can also avoid the ethical problems of ESC extraction from patients.^[^ ^86^ ^]^

In the last decade or so, with the breakthrough discovery of iPSCs, researchers have differentiated them into a variety of cells with different functions and phenotypes. iPSC gradually showed great potential for applications in the direction of drug testing, disease modeling, and regenerative medicine^[^ ^87^, ^88^ ^]^ (Figure 7D,E). However, these unidirectional differentiation techniques lack effective intercellular communication. iPSC often needs to receive guidance and regulation from neural cells, endocrine cells, related immune cells, and inflammatory cells. Due to their weak spontaneous differentiation ability, simply constructing in vitro stem cell models for disease modeling may be a great challenge. Therefore, we need to establish a more comprehensive and complex 3D culture system to meet the important conditions for multicellular spatiotemporal communication and finally complete sequential development.^[^ ^89^ ^]^ (Figure 7E).

**Figure 8:**
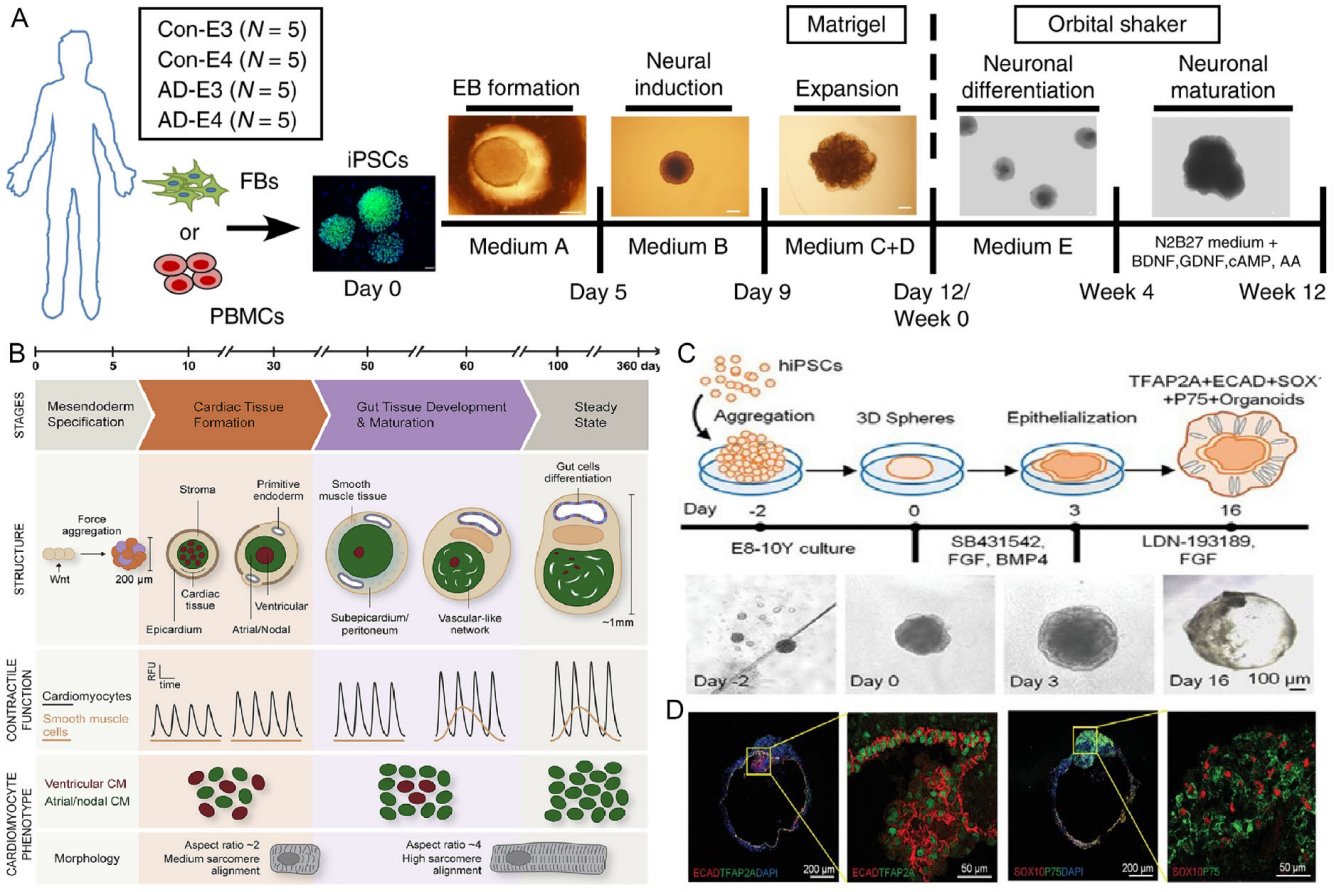
iPSC‐based organoid construction techniques have been applied to several disease areas. A) Schematic diagram of iPSC‐derived brain organoid construction. Reproduced under the terms of the CC‐BY Creative Commons Attribution 4.0 International license (https://creativecommons.org/licenses/by/4.0).^[^ ^90^ ^]^ Copyright 2020, The Authors, published by Springer Nature. B) Human multispectral iPSC‐derived cardiac and intestinal organoid. The presence of endodermal tissue (intestine) in the organoid helps the development of cardiac tissue characteristics. Reproduced with permission.^[^ ^91^ ^]^ Copyright 2021, Elsevier. C) Schematic diagram of iPSC‐derived mesenchymal (skin) organoid culture process. D) After 16 days, the organoid expressed epidermal markers (TFAP2A + ECAD +) and glial markers (SOX10 + P75 +). Reproduced under the terms of the CC‐BY Creative Commons Attribution 4.0 International license (https://creativecommons.org/licenses/by/4.0).^[^ ^92^ ^]^ Copyright 2022, The Authors, published by Wiley‐VCH.

As mentioned earlier, iPSC‐based organoids are constructed according to the developmental process. Therefore, iPSC‐derived organoids are often in the embryonic period and are more suitable for studying the state and physiology of early organs. Currently, iPSC‐based organoid construction techniques have been applied to several disease areas. Zhao et al.^[^ ^90^ ^]^ used iPSC with APOE ε3/ε3 or ε4/ε4 genotypes from normal individuals or Alzheimer's disease patients to construct brain organoids to study the potential pathogenic mechanism of APOE4 in Alzheimer's disease (**Figure** **8**A). Compared to healthy individuals, apoptosis was increased in Alzheimer's disease‐derived organoid brain tissue. Through 3D modeling, the team demonstrated that APOE4 exacerbates the accumulation of phosphorylated tau, which may be a new therapeutic target for the treatment of Alzheimer's disease. Silva et al.^[^ ^91^ ^]^ described a human multispectral iPSC‐derived heart and intestinal organoid (Figure 8B). The two distinct organs have distinct organizations and functions. Linking to the regulatory role of paracrine signaling during embryonic development, the team demonstrated that the presence of tissue from the mesoderm and endoderm (intestine) contributes to the development and maturation of cardiac tissue. This research progress helps to further explore the interactions between mature tissues and organs. Similarly, Ma et al.^[^ ^92^ ^]^ established human iPSC‐derived epithelial and mesenchymal (skin) organoid models in a 3D culture system (Figure 8C,D). The results showed that skin organoids enhanced the stem cell activity of the localized scleroderma and reduced the degree of skin fibrosis. This is a significant contribution to the treatment of skin diseases with skin organoids.

Although the technology of constructing soft tissue organoids is maturing, more and more soft tissue organoid models have been applied to various immune, inflammatory, and tumor diseases. However, the construction of bone organs is still in its infancy. In contrast to soft tissue organs, bone organs require precise regulation of the metabolism, mineralization, and deposition of bone matrix.^[^ ^93^ ^]^ Although there are many difficulties in the construction of bone organoids, many iPSC‐derived bone organoids have been birthed based on the bone reconstruction theory described in the previous section.

**Figure 9:**
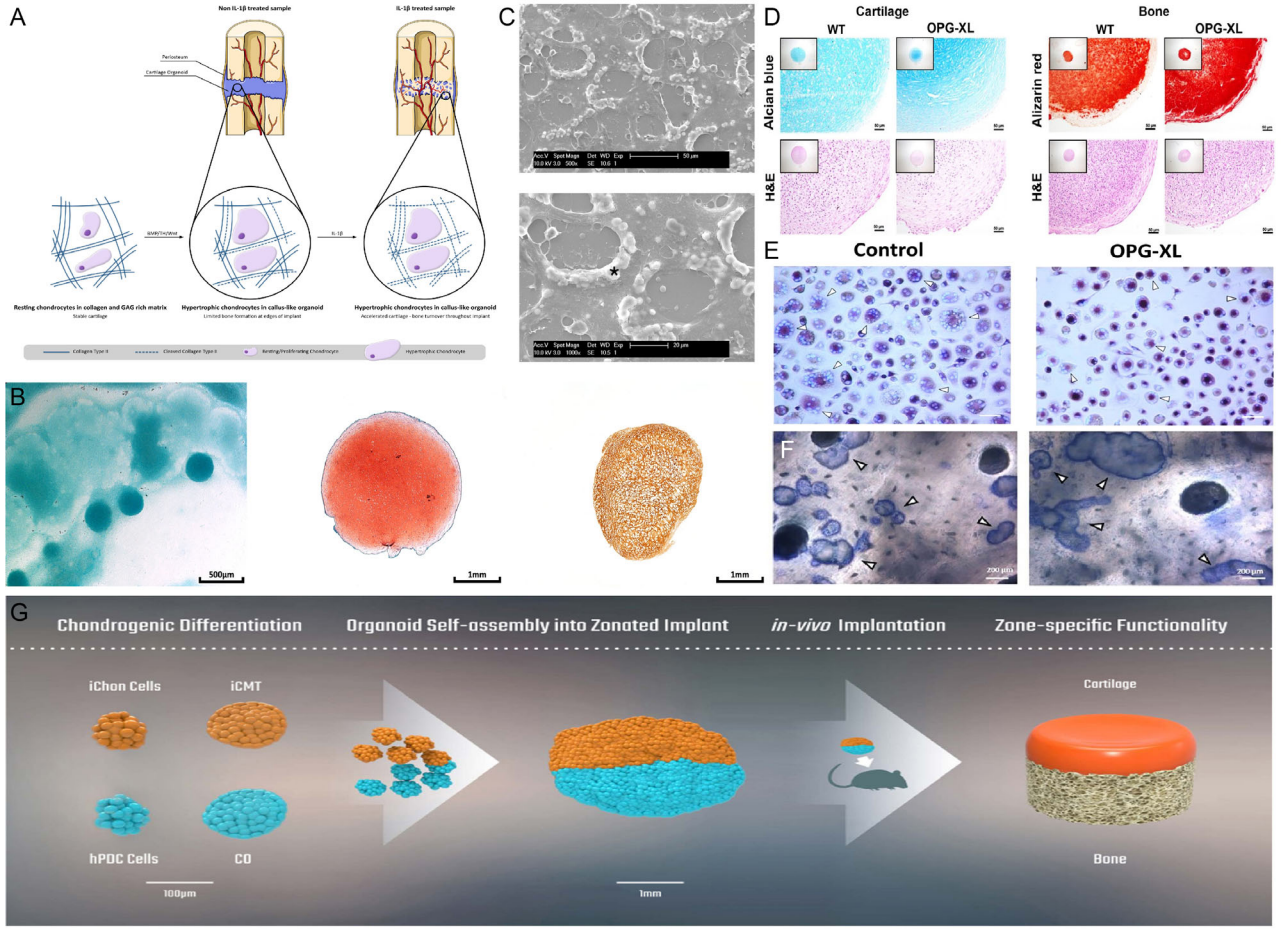
iPSC‐derived bone organoids. A) IL‐1β may accelerate bone healing by increasing the degradation of cartilage matrix by MMP13. B) Glycosaminoglycan‐rich nodules are detected at day 14. Positive staining for saffron O and type II collagen. C) Scanning electron microscopy shows white nodules around the chondrocytes. Reproduced under the terms of the CC‐BY Creative Commons Attribution 4.0 International license (https://creativecommons.org/licenses/by/4.0).^[^ ^94^ ^]^ Copyright 2021, The Authors, published by BMC, part of Springer Nature. D) Alcian blue, alizarin red, and H&E staining of new cartilage and new bone. E) Representative TRAcP (Tartrate‐resistant acid phosphatase) staining of osteoblasts after 21 days of culture with M‐CSF (macrophage colony‐stimulating factor) and RANKL. F) Representative images of bone resorption depressions formed by osteoclasts on human tibial sections after 21 days in culture. D–F) Reproduced under the terms of the CC‐BY Creative Commons Attribution 4.0 International license (https://creativecommons.org/licenses/by/4.0).^[^ ^95^ ^]^ Copyright 2022, The Authors, published by Oxford University Press on behalf of the British Society for Rheumatology. G) Cartilage from iPSC and hPDC forms a “callus organoids” assembly. Reproduced with permission.^[^ ^97^ ^]^ Copyright 2021, Elsevier.

Tam et al.^[^ ^94^ ^]^ used the time‐dependent nature of growth factors to adjust the culture conditions to culture human‐derived iPSC crust organoids in suspension in vitro. The engineered iPSC coalesced to form glycosaminoglycan‐rich clumps after mesoderm induction. Upon maturation, they gradually transform into type II collagen‐rich cartilage clumps. When implanted in situ into a critical bone defect, the bone organoid can recruit osteoblasts for repair and IL‐1β accelerates the bone repair process by increasing the degradation of the cartilage matrix by MMP13 (**Figure** **9**A–C).

Rodríguez Ruiz et al.^[^ ^95^ ^]^ constructed cartilage and bone organoids using human iPSC expressing OPG‐XL and the CRISPR/Cas9 system. Human OPG‐XL carriers were more likely to have a large number of active osteoblasts. The results also demonstrated that OPG‐XL expression leads to hyperfibrosis of cartilage and hypermineralization of the bone matrix. In turn, the articular cartilage calcification characterized by concomitant low subchondral bone mineralization is a hallmark of osteoarthritis pathophysiology. The mutated cartilage–bone analog of this gene also has the potential for future application to additional bone or cartilage degenerative diseases (Figure 9D–F).

O’Connor et al.^[^ ^96^ ^]^ developed bone analogs from mouse‐derived iPSC to investigate the interaction between bone and cartilage. Transforming growth factor β‐3 (TGF‐β3) and bone morphogenetic protein 2 (BMP2) were added sequentially in chronological order to stimulate the growth of single cells into organoids. This bone organoid model does not require multiple cell types and scaffold materials and has significant implications for the study of endochondral osteogenesis.

Hall et al.^[^ ^97^ ^]^ applied a bottom‐up strategy using a combination of cartilage from iPSC and human periosteum‐derived cells (hPDC) to form organoids (Figure 8G). Two different sources of cartilage microtissues can be engineered to form region‐specific cartilage implants. This bilayer structure mimics articular cartilage and subchondral bone, respectively. The design process, although more complex, was completed with a time‐dependent gradient developmental process through different culture conditions. This sequential differentiation of osteochondrogenic organoids has a greater potential to form osteochondral tissues with a hierarchical structure.

### 4.2 From Multidirectional Differentiated Adult Stem Cells to Bone Organoid

**Figure 10:**
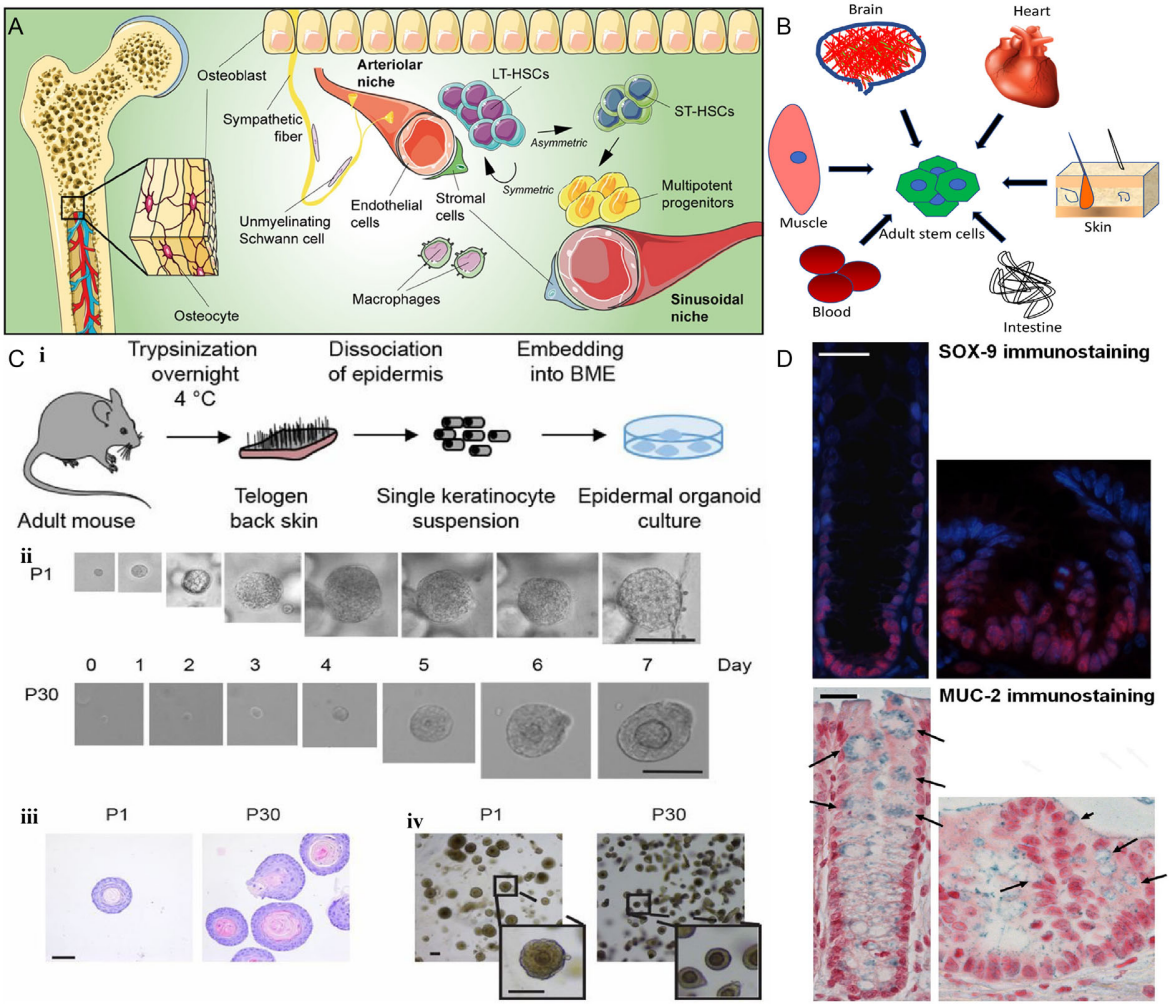
ASC‐derived bone organoids. A) Upon activation, LT‐HSC can generate ST‐HSC, which can produce pluripotent progenitor cells that can ensure normal hematopoiesis for up to 3 and 4 months. Reproduced with permission.^[^ ^99^ ^]^ Copyright 2022, The Authors, published by Wiley. B) Adult stem cells from different organs for regenerative therapy. Reproduced with permission.^[^ ^101^ ^]^ Copyright 2018, Elsevier. C) (i) Schematic diagram of mouse epidermal organoid construction. (ii) Changes in organoid morphology shown under light microscopy over a 7 day period after long‐term culture. (iii) HE staining images of the 1st and 30th generations after cell passage cultivation. (iv) Brightfield images of the first and thirtieth generations after cell passage cultivation. Reproduced with permission.^[^ ^105^ ^]^ Copyright 2019, The Authors, published by National Academy of Sciences, USA. D) Both SOX‐9 and MUC‐2 immunohistochemically positive cells were present in the transplanted structures 7 days after injection of colonic organoid cells. Reproduced with permission.^[^ ^106^ ^]^ Copyright 2020, The Authors, published by Sage Publications.

ASCs are undifferentiated cells present in an already differentiated tissue. They are capable of renewing themselves and differentiating into specific cells of the corresponding tissue or organ type.^[^ ^98^ ^]^ ASCs are present in various tissues and organs of the body and are mostly quiescent under normal conditions^[^ ^99^ ^]^ (**Figure** **10**A). ASCs are located at specific anatomical sites and are well protected from being disturbed or destroyed. As the basic cells for tissue homeostasis and regenerative repair, ASCs have the potential for multidirectional differentiation, thus maintaining a dynamic balance of growth and decline in tissues and organs. Due to low risk of tumorigenesis and lack of histocompatibility, ethical controversies and immune rejection are rare.

ASCs typically give rise to progenitor cells that have much less differentiation potential than embryonic stem cells. And they can only differentiate into specific cells of their tissue or organ, and their sources include bone marrow, peripheral blood, retina, skeletal muscle, skin, and intestine^[^ ^100^, ^101^ ^]^ (Figure 10B). Bone marrow mesenchyml stem cells (BMSCs) and hematopoietic stem cells (HSCs) were the first discovered adult stem cell populations, and their self‐renewal capacity and differentiation potential have been widely recognized.^[^ ^102^, ^103^ ^]^ Long‐term HSC (LT‐HSC) can produce more HSC upon activation, while short‐term HSC (ST‐HSC) can rapidly generate pluripotent progenitors to meet hematopoietic demand. When homeostasis is restored in vivo, HSC return to a quiescent state and retain a reversible self‐renewal capacity.^[^ ^99^ ^]^ Similarly, the relationship between the ecological microenvironment of BMSCs and tissue regeneration is extremely important. BMSCs are easily obtained and easily expanded in culture. In particular, the multidirectional differentiation potential of being able to differentiate in three directions, bone, cartilage, and fat, gives BMSC a central position in bone homeostasis and bone microenvironment.^[^ ^104^ ^]^

In contrast to iPSC, ASC‐derived organoids are mainly composed of mature cells, which tend to maintain high genetic stability after long‐term culture.^[^ ^86^ ^]^ However, the differentiation potential of ASC is inferior to that of iPSC, and the derived organoids are often only transformed into a single epithelial cell type. Boonekamp et al.^[^ ^105^ ^]^ used epithelial stem cells, determined optimal culture conditions, and successfully established a human epidermal organoid model (Figure 10Ci) with the promotion of cytokines such as fibroblast growth factor (FGF). The growth rate was approximately 7 days, regardless of the number of passages (Figure 10Cii–iv). This 3D system, which can be cultured for a long time, preserves the basal–apical tissue of the mouse interfollicular epidermis and is a good in vitro platform to study skin diseases. Moussa et al.^[^ ^106^ ^]^ cocultured colon stem cells and mesenchymal stem cells. The combined application of colon stem cells and MSCs improved intestinal radiation damage more than the colon organoids constructed from colon stem cells alone (Figure 9D). Irrespective of the type of ASC, their ability to differentiate in a directed single direction preserves the possibility of organoid construction.

As the most common stem cell type in bone or cartilage organoids, BMSCs have been widely used in bone remodeling mechanisms, osteoarthritis and osteoporosis. BMSCs have the ability to self‐renew and differentiate into a variety of cells and are therefore used as seed cells for tissue regeneration and stem cell therapy. BMSCs are derived from bone marrow and the direction of differentiation depends on the microenvironment in which they are located. In BMSCs, osteogenesis and adipogenesis are thought to be mutually exclusive differentiations. Maintaining the balance of BMSCs differentiation is essential for skeletal homeostasis. In addition to their strong value‐added capacity and multidirectional differentiation potential, BMSCs can also modulate immune function and regulate cellular interactions.^[^ ^107^ ^]^ Surface antigens are less pronounced in BMSC and graft rejection is less severe, making them the cell type of choice for bone or cartilage‐based organ transplantation therapy. BMSCs have been shown to have some shortcomings: for example, the ability of the cells to proliferate and differentiate decreases significantly with age, and the number of cells extracted is limited. As stem cells cohabiting in the bone marrow microenvironment, HSCs ensure the homeostasis of progenitor cells and the hematopoietic system through self‐renewal, differentiation, and migration.^[^ ^108^ ^]^ Macrophages and osteoclasts can regulate HSCs through several mechanisms, and osteoblasts, although they cannot directly regulate HSCs, can indirectly influence the function of HSCs by secreting cytokines that crosstalk with other cells in the bone marrow.^[^ ^109^ ^]^ Thus, it can be seen that in the bone marrow microenvironment, bone marrow cells such as HSCs, BMSCs, osteoblasts, osteoclasts, and macrophages interact with each other through a variety of regulatory factors to participate in hematopoiesis, osteogenesis, repair, and ecological maintenance functions.

**Figure 11:**
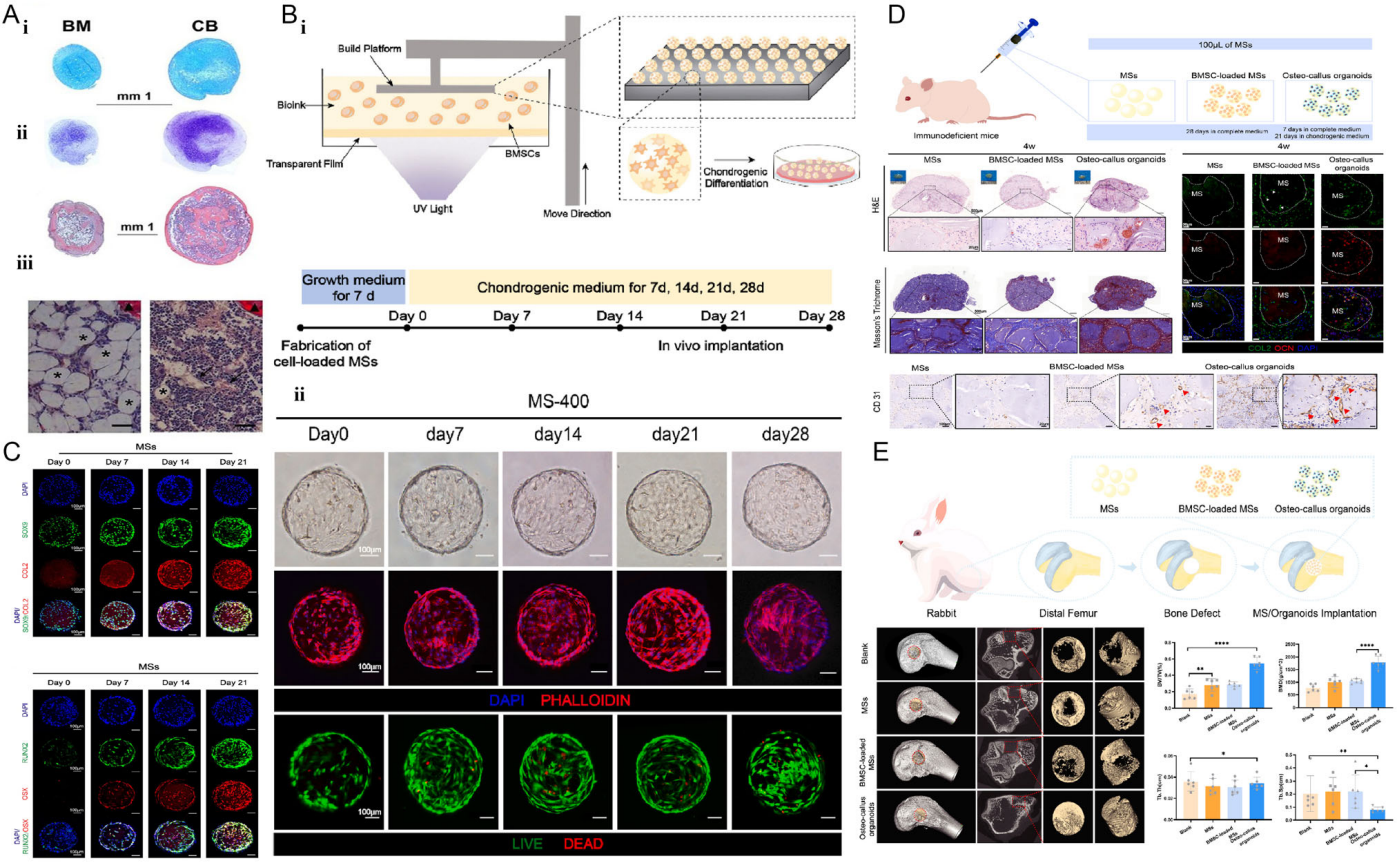
Construction of CB‐BFs and BMSCs‐derived bone organoids. A) (i) Representative histological images of cartilage organoid obtained from BMSCs (left) and CB‐BFs (right) (top, Alcian blue; bottom, toluidine blue; ii, HE). (iii) Representative histological images of bone marrow tissue (Sirius red staining) in small bones produced from cartilage organoid from BMSCs (left) or CB‐BFs (right) after transplantation are shown. Reproduced with permission.^[^ ^110^ ^]^ Copyright 2017, The Company of Biologists. B) (i) Construction of BMSC‐based organoid by 3D printing (complete growth medium for 7 days and cartilage differentiation medium for 28 days). (ii) Up, representative bright field images. Middle, Confocal z‐projection images of phalloidin/DAPI staining. Bottom, live/dead staining of BMSCs cultured in microspheres without induction (day 0), at day 7, day 14, day 21, and day 28. C) Representative confocal z‐projection images of SOX9 and COL2 (up) or RUNX2 and OSX (bottom) immunofluorescent staining of BMSCs in microspheres without induction (day 0), at day 7, day 14, and day 21 of chondrogenic induction. D) In vivo implantation of osteocallus organoids. E) Micro‐CT evaluation of new bone formation 4 weeks after implantation in a rabbit distal femoral bone defect model. B–D) Reproduced with permission.^[^ ^111^ ^]^ Copyright 2022, Elsevier.

Pievani et al.^[^ ^110^ ^]^ developed an in vivo model using material derived from human umbilical cord blood (CB). Cord blood‐derived fibroblasts (CB‐BFs) formed cartilage‐like organs in vitro and were later transplanted into mice. Histomorphometric analysis and the number of hematopoietic cells isolated within the bone of CB‐BFs demonstrated their superior hematopoietic potential compared to the organoid formed by BMSCs. The team also evaluated the osteogenic potential of CB‐BFs and verified that they are an osteoblast with specific histologic characteristics (**Figure** **11**A). CB‐BFs have osteogenic and organoid building abilities similar to those of ASCs.

Xie et al.^[^ ^111^ ^]^ loaded BMSCs onto hydrogel microspheres by Digital Light Processing (DLP) printing and distribution induction (Figure 11B). The aggregates of BMSCs alone limited the further development of endogenous osteogenesis, whereas osteocallus organoids showed strong bone regeneration potential. Osteocallus organoids, into which BMSCs were transformed after chondrogenesis induction, were able to recapitulate the endochondral osteogenesis process (Figure 11C). In vivo experiments also showed that osteocallus organoids were able to repair bone defects in rabbits within 4 weeks, significantly shortening the time to bone healing (Figure 11D,E).

**Figure 12:**
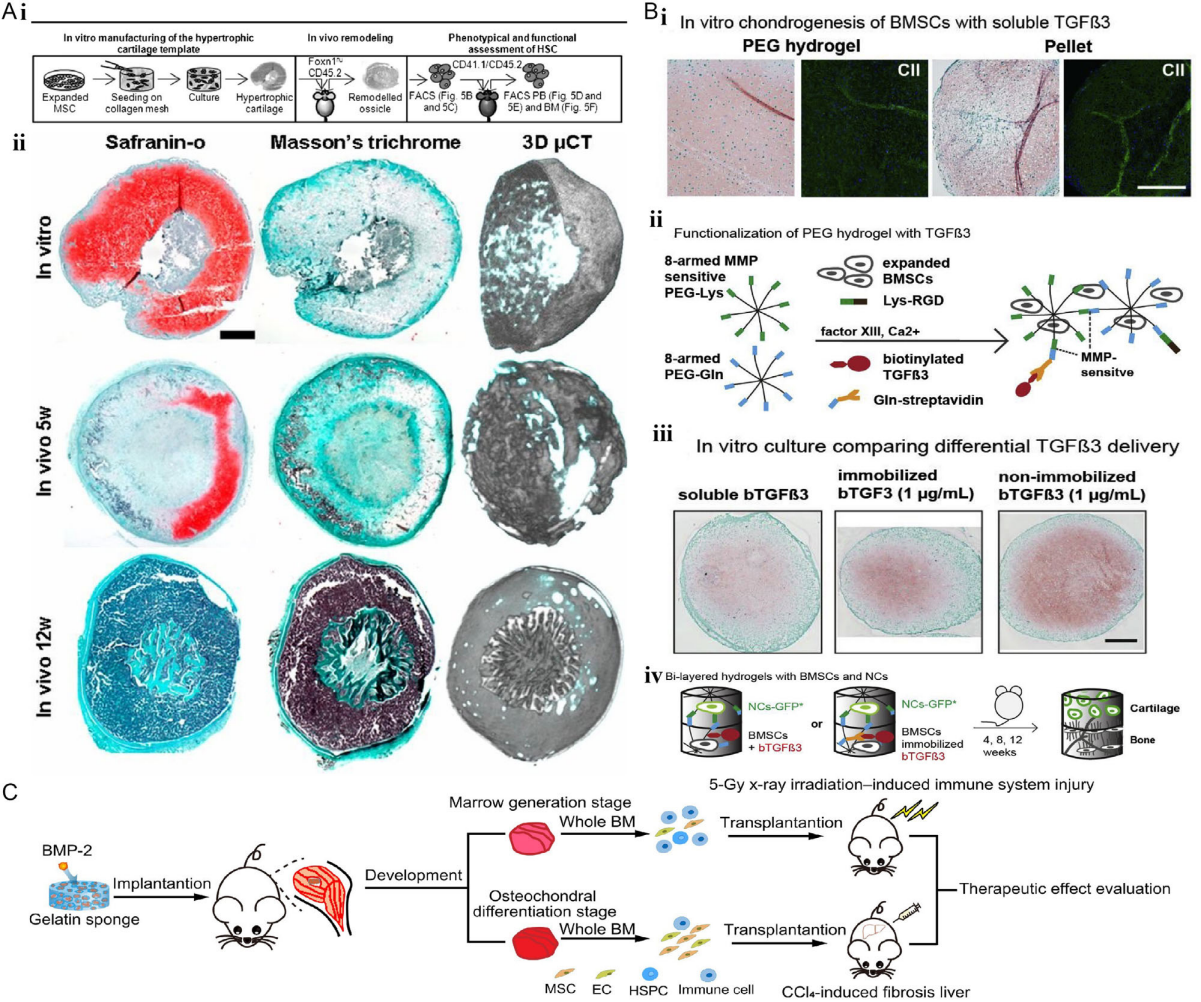
Cartilage‐based bone organoids. A) (i) Schematic diagram of in vitro synthesis to in vivo implantation. (ii) Safranin O, Masson trichrome section staining, and 3D reconstructed microtomography images of samples cultured in vitro for 5 weeks and ectopically implanted in nude mice at 5 and 12 weeks. Reproduced with permission.^[^ ^112^ ^]^ Copyright 2013, The Authors, published by National Academy of Sciences, USA. B) (i) BMSCs were encapsulated in PEG hydrogel containing RGD peptides or prepared into cell spheres. Type II collagen, safranin O‐fast green, and immunofluorescence staining. (ii) Immobilization was achieved by covalently binding streptavidin (Strep) and biotinylated TGF‐β3 in a hydrogel network. (iii) Safranin O‐fast green images of BMSCs cultured in immobilized or unimmobilized TGF‐β3 0.5, 1, and 3 μg mL^−1^ and soluble TGF‐β3 hydrogels for 2 weeks. (iv) BMSCs were encapsulated in a hydrogel containing immobilized or nonimmobilized TGF‐β3 and placed on another layer containing nasal cartilage without TGF‐β3 and implanted directly subcutaneously into nude mice. Reproduced with permission.^[^ ^113^ ^]^ Copyright 2018, Elsevier. C) Implantation of gelatin sponge containing BMP‐2 into the medial muscle pocket near the femur of mice to produce bone organoid. Reproduced with permission.^[^ ^114^ ^]^ Copyright 2023, The Authors, published by American Association for the Advancement of Science.

Scotti et al.^[^ ^112^ ^]^ developed an endochondral osteogenesis model using a developmental engineering strategy. BMSCs were first inoculated onto a collagen scaffold as a template for development (**Figure** **12**A). IL‐1β was then added to the culture medium to promote cartilage remodeling. The BMSC‐based cartilage organoid can perform important physiological functions, including bone reconstruction, angiogenesis, and hematopoiesis. In addition, the medullary cavity of the organoid contains HSCs and various types of progenitor cells similar to those of natural bone, and the structure and function are also comparable to those of natural bone. This organoid provides a useful model for basic studies of bone morphogenesis and regulatory mechanisms of HSCs

Stüdle et al.^[^ ^113^ ^]^ pioneered the creation of a cartilage–bone bilayer organoid capable of autonomous and orderly development (Figure 12B). Briefly, the team embedded BMSCs and nasal chondrocytes in a bilayer hydrogel. The bilayer hydrogel consisted of a TGFβ3‐ or BMP‐2‐functionalized polyethylene glycol hydrogel (encapsulated BMSCs) and a hydrogel containing nasal cartilage. The BMSCs‐loaded hydrogel layer allows for endochondral osteogenesis and the nasal cartilage hydrogel layer allows for cartilage tissue formation. This bilayer structure facilitates the process of mimicking the development of the cartilage–bone interface and also helps to repair joint injuries.

Dai et al.^[^ ^114^ ^]^ explored the potential application of bone organoid containing BMP‐2 scaffolds for the treatment of bone defects at different developmental stages. This cell‐free strategy serves as an ecological environment for stem cells and immune cells to communicate and exchange, and can harvest abundant autologous cells for osteogenesis, fibrogenesis, and hematopoiesis (Figure 12C). The organoids are able to rapidly reconstitute the immune system during three interacting phases: fibrogenesis, chondrogenic differentiation, and myelopoiesis. In addition, this type of organoid is able to recruit stem cells for a good regenerative repair task.

### 4.3 Alternative Cellular Strategies for Bone Organoid Construction

Stem cells, as the most primitive undifferentiated cells, are an important component of organoid construction because of their potential multidirectional differentiation. Periosteum‐derived cells (PDCs) are expected to provide a new source of stem cells for regenerative medicine.^[^ ^115^ ^]^ Multiple cells are often involved in different stages of bone or cartilage repair. In the bone marrow or local microenvironment of the defect, the process of osteoblast and osteoclast commitment, migration, and phagocytosis predominate. Osteoblasts are responsible for new bone formation, matrix synthesis, and mineralization, while osteoclasts are responsible for degradation and resorption of old bone. When the balance between new and old bone is disturbed, abnormalities in bone structure or function occur, such as delayed fracture healing and osteoporosis. Multiple cytokines and signaling pathways are involved in bone remodeling. Osteoblasts and osteoclasts can also communicate through direct cell‐to‐cell contact or indirect cytokine‐to‐cell communication.^[^ ^116^ ^]^ The bidirectional action of osteoblasts and osteoclasts is essential for maintaining bone homeostasis in vivo.^[^ ^117^ ^]^ The development of novel bone organoid is also gradually shifting to stem cells and relying on mature osteoblasts or osteoclasts.

**Figure 13:**
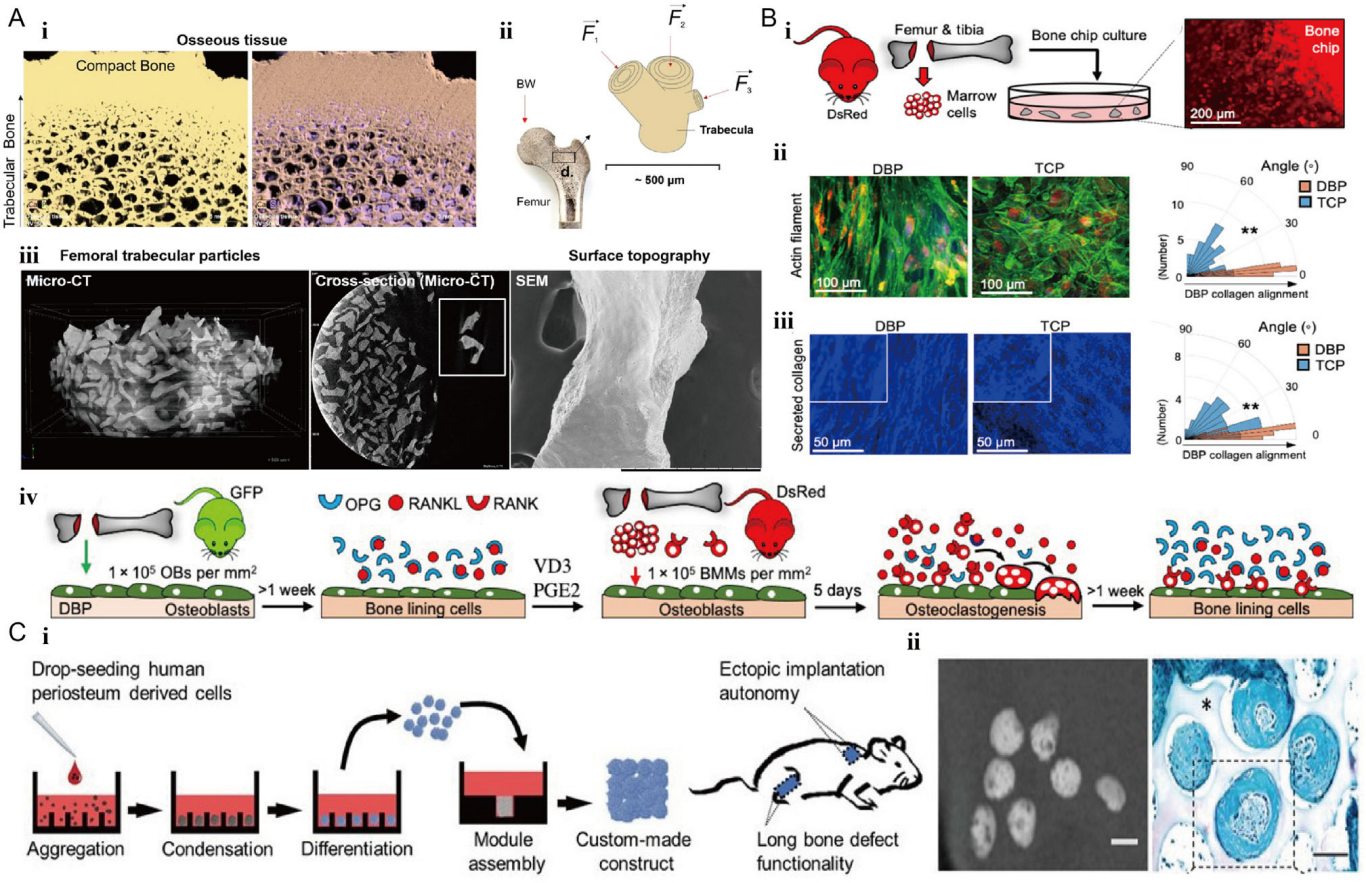
Bone organoids based on osteoblasts and osteoclasts. A) (i) This compact trabecular structure is high in calcium phosphate (left) and low in protein (right). (ii) Each trabecular bone is subjected to forces from multiple directions. (iii) Analysis of bone trabeculae granularity, laminar structure and morphology. Reproduced under the terms of the CC‐BY Creative Commons Attribution 4.0 International license (https://creativecommons.org/licenses/by/4.0).^[^ ^93^ ^]^ Copyright 2021, The Authors, published by Springer Nature. B) (i) Osteoblasts obtained from DsRed mice. (ii) Immunofluorescence staining of actin filaments of osteoblasts (left) and circular histogram of cell alignment angle (right). (iii) Multiphoton second harmonic (SHG) image (left) and circular histogram of collagen fiber alignment angle (right). (iv) Schematic diagram of the experiment simulating the bone reconstruction cycle. Reproduced with permission.^[^ ^118^ ^]^ Copyright 2021, The Authors, published by American Association for the Advancement of Science. C) (i) Schematic diagram of the bioengineering process starting from cell aggregation, condensation, and differentiation. (ii) Nano‐CT images and Safranin O images 4 weeks after in vivo implantation. Reproduced under the terms of the CC‐BY Creative Commons Attribution 4.0 International license (https://creativecommons.org/licenses/by/4.0).^[^ ^119^ ^]^ Copyright 2019, The Authors, published by Springer Nature.

Iordachescu et al.^[^ ^93^ ^]^ pioneered the new concept of micrometer‐scale bone organoids. The model consists of a combination of osteoblasts and osteoclasts that produce relevant phenotypes and functions at the cell–tissue interface (**Figure** **13**A). This organoid is seeded on femoral bone trabeculae and serves as a multicellular organ model that can well simulate the bone loss process. Under natural conditions, there is a regulatory balance between osteoblasts and osteoclasts with respect to mechanical stimulation, hormonal or small molecule protein stimulation. The model can effectively provide an ex vivo platform to overcome the limitations of traditional laboratory simulation of the bone microenvironment.

Park et al.^[^ ^118^ ^]^ constructed a bone organoid with high fidelity and controllability. The team used an osteoblast‐like material to support osteocytes to guide the structural mineralization of osteoblasts (Figure 13B). This human‐derived trabecular organoid can significantly improve clinical prediction and shorten the screening time for osteoporosis drugs. While elucidating the mechanisms of bone metabolism, this model can also be used to study the quiescence and activation of bone marrow hematopoietic stem cells.

Nilsson Hal et al.^[^ ^119^ ^]^ used PDCs to create an artificial bone organoid for the regenerative treatment of long bone defects (Figure 13C). During natural fracture healing, most of the cells in the bone scab originate from the periosteum.^[^ ^120^ ^]^ PDCs, as stem cells present in the periosteum, have the same or even greater repair capacity than BMSCs. The results showed that both the time of new bone formation and the proportion of bridging with old bone were highly similar to natural long bone. Importantly, the in vitro maturation and development process of this model is influenced by the gene expression pattern. Even in vitro, the natural cascade process is simulated. This revolutionary result will pave the way for the future production of clinical implants.

## 5 Direction of Application of Stem Cell and Biomaterial‐Based Bone Organoid

### 5.1 Construction of Regenerative Models

The goal of regenerative therapy is to repair organs with poor function or structure. In an immunosuppressed state, it reduces graft toxicity, improves quality of life, and maximizes the harm and inconvenience caused by organ or tissue loss.^[^ ^121^ ^]^ Currently, both autologous and allogeneic bone grafts are often limited in clinical use due to rejection or donor shortage. The recent emergence of biomaterial grafts may address some of these limitations, but the physiological response and subsequent status of the graft in the recipient requires rigorous preclinical evaluation. In addition, conventional tissue engineering can only replace a few functions and structures of bone, which also severely limits the effectiveness of bone regeneration and repair.^[^ ^122^ ^]^

The bone organoid is a 3D in vitro culture system with a more complete range of cell types and extracellular environments. Bone organoids could highly simulate the real location and spatial morphology of cells and matrix in vivo, and also clearly show the results of cell–cell and cell–matrix interactions. The mechanisms of bone regeneration are more complex, involving multiple steps and multiple substances. Traditional models of bone defects (fractures), osteoarthritis, osteoporosis, and bone tumors are commonly used in animal experiments in rats, rabbits, or goats. When relying solely on in vivo models, there is variability in experimental results and the difficulty and cost of experimentation are much greater. Bone organ materials are widely available and easy to obtain. They can maintain a stable phenotype and function in vitro and can expand or differentiate autologously, mimicking the bone regeneration process in all aspects. In addition, bone organoid can produce and utilize relevant cytokines or other bioactive components to indirectly or directly induce osteogenesis and accelerate bone regeneration.

Regenerative medicine heralds a new era of reconstruction, regeneration, “manufacturing,” and replacement of tissues and organs, offering new hope for most of the medical problems facing humanity. From a clinical perspective, there is an urgent need for a regenerative graft that meets the requirements of tissue transplantation both structurally and functionally. The cellular origin of bone organoids is the first consideration. Regenerative medicine takes advantage of the multidirectional differentiation properties of stem cells, which can achieve osteogenic induction and bone tissue regeneration with the aid of a vector. Second, bone organoids with a similar composition to ECM could also be a good substitute for defective site tissue. For bone organoids, the availability of mature bioactive materials and abundant sources of stem cells will facilitate the choice of clinical treatments.

### 5.2 Drug Testing and Screening

Conventional bone tissue engineering scaffolds are rarely used for drug testing and screening due to limitations such as long cycle time, many interferences, and high cost. The cell lines or primary cells grown in artificial culture environment alone are not sufficient to meet the multiple cells and information transfer required for drug testing. Common drugs for orthopedic diseases such as bisphosphonates, celecoxib, diclofenac, and glucosamine for antimetabolism, pain relief, and cartilage protection require effective preclinical testing in view of different age groups and indications.^[^ ^123^ ^]^ For bone organoids, hydrogels with close or identical physicochemical properties to those in vivo are used to avoid the rigid mechanical environment of 2D culture.^[^ ^124^ ^]^ For the requirements of long‐distance cell communication, direct cell‐to‐cell contact or immune cell phagocytosis and delivery required for toxicological assays, the miniature multicontent model of bone organoid coculture has excellent performance under various pathophysiological conditions. As primary cells, iPSC‐based bone organoids not only have the ability to self‐renew, but also facilitate the expansion of drug screening. Bone analogues can shorten the screening cycle and provide a resource for drug toxicity analysis and individualized therapy.^[^ ^125^ ^]^ The results of 2D flat culture often contradict the actual condition of the patient. Compared to animal models, smaller and more accurate organoid drug screening models are more suitable.

### 5.3 Evaluation of Implant Materials

Bone biomaterials are bridges between native and neoplastic tissues and play a central crosstalk role in bone regeneration and repair.^[^ ^126^ ^]^ Reactions such as chemical bond formation and tissue resorption often occur between biomaterials and surrounding tissues.^[^ ^127^ ^]^ Biomaterials with osteoinductive properties can modulate the rate and process of bone and cartilage regeneration. To better achieve a structure and function similar to natural bone, we need to evaluate the fundamental physicochemical properties of the material from multiple perspectives and in all aspects. Currently, in vitro cellular experiments and in vivo animal experiments are the two mainstream evaluation tools for biological implant materials. The evaluation of cellular experiments alone has the same limitations as drug screening in the previous section. A single cell type and a small number of cellular communications do not provide strong convincing evidence for preclinical testing. Animal studies often require consideration of constraints such as feed costs, housing, environmental conditions, ease of animal acquisition, and societal ethical requirements.^[^ ^128^ ^]^ Sometimes, species differences and batchiness of data can also influence the best evaluation results. In contrast, bone organs provide a simple but comprehensive platform for research. Not only does it simulate the real in vivo environment and reproduce physiological processes such as cellular communication, but it also allows the direct use of human‐derived cells to avoid applicability errors due to species differences.

### 5.4 In Vitro Culture for Regrafting

As mentioned earlier, autologous or allogeneic bone grafts suffer from problems such as donor shortage. Conventional biomaterials and even tissue‐engineered scaffolds have limitations, such as inadequate matching with natural bone for implantation. As an emerging tool for in vitro culture–in vivo implant evaluation, bone‐based organs are also emerging as effective implants for the treatment of large segmental bone defects. BMSC‐ or iPSC‐based bone or cartilage organoids are often constructed in vitro for human bone defects or diseases such as osteoarthritis. Hyaluronic acid hydrogel, which is beneficial to the articular environment, is also used as an extracellular matrix. After osteogenic or chondrogenic differentiation, the complete 3D spheroid model is implanted into the defect site. The cell type and environment are similar or identical to the natural bone microenvironment, avoiding immune rejection and allowing for sequential growth and development. This artificial bone scab meets the requirements of fracture treatment guidelines, maximizing cellular activity and stimulating osteogenic potential. Unfortunately, this field is in early stages and has not developed sufficiently to produce actual organs.

## 6 Summary

Bone organoid is a concept that has emerged in the context of the bottleneck of traditional bone tissue engineering research. Bone organoids use various nontoxic matrix materials and various types of stem cells for in vitro 3D culture to induce cell‐directed differentiation to achieve self‐assembly, self‐renewal, and self‐communication after maximizing the simulation of natural bone structure and microenvironment. Although there is no bone organoid that meets the standard definition, a design strategy based on cellular self‐forming still conceptually supports the definition. The primary task of bone organoids is to promote osteogenesis and complete regenerative repair or in vitro evaluation. Osteogenesis is a complex and continuous process involving histologic and cytologic changes at all stages. A single cell type or culture environment cannot provide a complex pathological state. It is the full spectrum of cell–cell and cell–matrix communication that together orchestrate the regenerative repair processes of inflammatory response, osteogenic differentiation, and bone scab remodeling in vivo.

The strong self‐organizing ability of human cells is the cellular basis for in vitro organoid construction. Currently, common bone organoids use stem cells as the main cell type. Stem cells can generate the desired progeny cells with the help of growth factors and matrix while self‐organizing into 3D structures. Stem cells can be divided into ESCs and ASCs, depending on the sequence of individual development. ESCs are abundant and have a high proliferative capacity. Although ESCs are totipotent, stem cells of allogeneic origin may cause immune rejection. The construction of bone organoids from ESCs may require long‐term adjuvant therapy with immunosuppressive agents. There is also a risk of failure to induce normal gene expression when using clonogenic construction strategies based on autologous ESCs. ASCs have less differentiation potential than ESCs, but can differentiate into cells specific to the tissue in which they reside. Although ASCs have the ability to differentiate into multiple lineages, the “efficiency” of this differentiation is not yet optimal. The efficiency of transformation can be improved by in vitro expansion culture, but it remains to be proven whether in vitro transformation causes genetic changes in ASCs. The use of ESCs to construct bone organoids is the most desirable approach, but it has not progressed well due to ethical constraints such as embryo extraction. In contrast, ASCs, despite their limited ability to differentiate, are relatively convenient for research and are currently the best cellular option for a bone organoid construction strategy.

iPSCs and BMSCs are the two most common types of basic stem cells for building bone organoids. iPSCs are widely available, easy to cultivate, and can achieve multidirectional differentiation and self‐expansion. It also avoids ethical issues. BMSCs are derived from bone marrow, homologous to the implantation site, and have a greater capacity for osteogenesis, chondrogenesis, and lipogenesis in a more detailed way. The presence of stem cells facilitates the steps of regenerative repair. Bone organoids also have a much higher survival rate after implantation because they carry this type of seed cells. The use of osteoblastic and osteoclastic cells alone is also a proven way to build bone organoids compared to stem cells, especially in terms of osteoclast mineralization. Bone organoids not only serve as a platform for in vitro simulation evaluation, but also as implants for the treatment of orthopedic diseases, which is an integrated treatment modality that integrates evaluation, screening, and treatment.

Finally, the process of bone mineralization of bone organoids in vitro is inseparable from the assembly of the ECM. Although cells can self‐organize into clumps, spheres, or organ shapes, they often cannot fully function without the protection, regulation, and fusion of the matrix. And ECM contains a variety of cytokines that contribute to cell proliferation and differentiation as an important part of bone ecology. The ECM is an embedded network of cells that not only provides physical support but also participates in the division and migration of cells within it. The current sources of ECM for bone organoids are mainly matrix gels, natural hydrogels, synthetic hydrogels, and decellularized matrices. These materials mimic the true ECM composition and function to varying degrees. Matrix materials can encapsulate various stem cells, protect, and support cell growth and development. The excellent physiological properties of matrix materials will help bone organoids shine in areas such as drug testing and in vivo implantation.

## 7 Outlook, Challenges, and Conclusions

As the most promising alternative platform for cellular and animal testing in orthopedic research, bone organoids are advancing at a rapid rate. Bone organoids reveal the repair mechanisms of human orthopedic degenerative and traumatic diseases and present remarkable features of human bone and cartilage development and growth. In recent years, other fields such as gastroenterology have brought organoid technology into the clinic. The use of organoids can accurately predict the pharmacological response and side effects of drugs in order to target and develop personalized treatment plans. In the field of orthopedics, especially in trauma, the construction and implantation of organoid models of bone defects can greatly assist clinicians in repairing large bone defects. Compared to conventional tissue‐engineered scaffolds, bone organoids derived from primary human cells have better biocompatibility. In the field of osteoarthritis, organoids may also gradually replace traditional cellular therapies. In addition, the addition of inorganic components such as calcium or phosphorus and programmed mineralization processes also influence the maturity and function of bone organoids. Hyaluronic acid and chondrocyte‐based organoids are expected to revolutionize the field of orthopedic regeneration. Nevertheless, there are still some problems with bone analogues that deserve our attention and solution in the future:

*Poor vascularization of bone organoids*: Fracture healing requires a large number of blood vessels to ensure the transport of nutrients and metabolic waste products. Lack of blood supply often results in local necrosis of bone and cartilage tissue, leading to delayed or nonhealing. The commonly used stem cells and osteoblasts can only expand and differentiate in the osteogenic direction and cannot meet the high requirements of vascularization. In the future, it would be useful to add host vascular endothelial cells and related growth factors to induce angiogenesis during the culture process. Alternatively, the organoid system can be integrated into the host vasculature to achieve relative stability in the extracellular environment.

*Lack of multisystem cooperation in bone organoids*: Although bone organoids can effectively mimic the responses of osteogenesis, osteoclastic resorption, matrix deposition, and inflammatory regulation in vitro, the mode of differentiation remains localized to the skeletal system. Actual bone defect healing is a multisystemic regulatory process involving the entire body. For example, the nervous and endocrine systems regulate physiological processes such as calcium and phosphorus levels, macrophage migration, and capillary reopening through neurotransmitters and hormones. The digestive, cardiovascular, and renal systems are involved to varying degrees in the pathophysiologic stress and late self‐healing processes of fracture. The bone organoids are still in the primary stage of a single system, and the model as a whole lacks neural and other distributions. There may be limitations in the results of drug testing or implant activity testing.

*Bone organoids are less reproducible*: As a stem‐cell‐derived model, its intrinsic cell fate is determined by a combination of factors. Both the shape and size of the organoid as well as its maturation and expansion capacity are highly heterogeneous. Even if the culture conditions are consistent and the cells are of the same origin, it is difficult to standardize mass production due to a variety of uncontrollable factors such as cytokines, cell number, or physicochemical properties of the matrix. This greatly limits the use of bone analogues in drug toxicology analysis and modeling of orthopedic diseases.

*Lack of immune cells in bone organoids*: Although orthopedic diseases are less dependent on immunotherapy, there is some immune regulation of fractures and arthritis due to the complexity and size of the human immune system. Common immune cells, such as T cells, require sites of maturation and peripheral circulation. Bone organoids cannot fully support the formation of the immune system due to the single system and fixed source of cells.

*Bone organoids are limited in size*: Common methods for organoid fabrication include manual mixing of matrix gels and cell suspensions, or centralized aggregation using bioreactors. Due to the maintenance of spherical morphology, the nutrient and oxygen content is not uniformly distributed inside and outside of spheroid organoids, which often leads to internal hypoxia and necrosis. Or the lack of nutrients affects the progressive differentiation and maturation process, leaving the organoid size at the millimeter level and below. In the future, new fabrication methods must be developed to enable more precise cell deposition and self‐organization.

In summary, bone organoids mimic the 3D structure and dynamic function of bone. It simplifies the culture cycle while maintaining the stability of the system. It has a wide range of applications in regenerative medicine and drug testing. Despite the current limitations, bone organoids are still a powerful tool for various studies. In the future, bone organoids will move from basic research to clinical applications, bringing unlimited opportunities for human orthopedic diseases.

## Conflict of Interest

The authors declare no conflict of interest.
